# Integrated proteomics identifies PARP inhibitor-induced prosurvival signaling changes as potential vulnerabilities in ovarian cancer

**DOI:** 10.1016/j.jbc.2022.102550

**Published:** 2022-09-29

**Authors:** Ou Deng, Sweta Dash, Thales C. Nepomuceno, Bin Fang, Sang Y. Yun, Eric A. Welsh, Harshani R. Lawrence, Douglas Marchion, John M. Koomen, Alvaro N. Monteiro, Uwe Rix

**Affiliations:** 1 Department of Drug Discovery, H. Lee Moffitt Cancer Center & Research Institute, Tampa, Florida, USA; 2 Department of Cancer Epidemiology, H. Lee Moffitt Cancer Center & Research Institute, Tampa, Florida, USA; 3 Proteomics & Metabolomics Core, H. Lee Moffitt Cancer Center & Research Institute, Tampa, Florida, USA; 4 Chemical Biology Core, H. Lee Moffitt Cancer Center & Research Institute, Tampa, Florida, USA; 5 Biostatistics and Bioinformatics Shared Resource, H. Lee Moffitt Cancer Center & Research Institute, Tampa, Florida, USA; 6 Tissue Core, H. Lee Moffitt Cancer Center & Research Institute, Tampa, Florida, USA; 7 Molecular Oncology, H. Lee Moffitt Cancer Center & Research Institute, Tampa, Florida, USA

**Keywords:** ADP-ribosylation, protein complex, DNA damage response, AKT, mTOR, ovarian cancer, DDR, DNA damage response, DMSO, dimethyl sulfoxide, FDR, false discovery rate, MS, mass spectrometry, NHEJ, nonhomologous end joining, OC, ovarian cancer, PTM, posttranslational modification, TMT, tandem mass tag

## Abstract

*BRCA1/2*-deficient ovarian carcinoma (OC) has been shown to be particularly sensitive to poly (ADP-ribose) polymerase inhibitors (PARPis). Furthermore, *BRCA1/2* mutation status is currently used as a predictive biomarker for PARPi therapy. Despite providing a major clinical benefit to the majority of patients, a significant proportion of *BRCA1/2*-deficient OC tumors do not respond to PARPis for reasons that are incompletely understood. Using an integrated chemical, phospho- and ADP-ribosylation proteomics approach, we sought here to develop additional mechanism-based biomarker candidates for PARPi therapy in OC and identify new targets for combination therapy to overcome primary resistance. Using chemical proteomics with PARPi baits in a *BRCA1*-isogenic OC cell line pair, as well as patient-derived *BRCA1*-proficient and *BRCA1*-deficient tumor samples, and subsequent validation by coimmunoprecipitation, we showed differential PARP1 and PARP2 protein complex composition in PARPi-sensitive, *BRCA1*-deficient UWB1.289 (UWB) cells compared to PARPi-insensitive, *BRCA1*-reconstituted UWB1.289+BRCA1 (UWB+B) cells. In addition, global phosphoproteomics and ADP-ribosylation proteomics furthermore revealed that the PARPi rucaparib induced the cell cycle pathway and nonhomologous end joining (NHEJ) pathway in UWB cells but downregulated ErbB signaling in UWB+B cells. In addition, we observed AKT PARylation and prosurvival AKT-mTOR signaling in UWB+B cells after PARPi treatment. Consistently, we found the synergy of PARPis with DNAPK or AKT inhibitors was more pronounced in UWB+B cells, highlighting these pathways as actionable vulnerabilities. In conclusion, we demonstrate the combination of chemical proteomics, phosphoproteomics, and ADP-ribosylation proteomics can identify differential PARP1/2 complexes and diverse, but actionable, drug compensatory signaling in OC.

Ovarian cancer (OC) is a highly lethal cancer that is often diagnosed at advanced stages, which leads to a low 5 year survival (1). Dissecting the biological roles of BRCA1 and BRCA2 proteins has led to understanding their central role in the DNA damage response (DDR) and laid the ground for the discovery of synthetic lethality approaches with poly (ADP-ribose) polymerase 1 (PARP1) inhibitors for tumors with pathogenic variants of *BRCA1* and *BRCA2* (2, 3, 4). Unlike normal cells, *BRCA1/2*-deficient cancer cells critically depend on PARP1 for backup DNA repair leading to selective killing of these cancer cells by targeting PARP1 and recent regulatory approval of several PARP1 inhibitors (PARPis) for *BRCA1/2*-linked OC (5, 6, 7, 8, 9). Although PARPis produce major response rates of 40% to 60% for patients with *BRCA1/2*-linked advanced OCs, a significant number of tumors does not respond to these drugs (5, 10, 11, 12). While multiple studies have identified mechanisms of acquired resistance to PARPis, the processes that govern this primary resistance are incompletely understood. Although a recent study reported pre-existing *BRCA*-reversion mutations in a small subpopulation of patients as a consequence of prior platinum-based chemotherapy (13), the majority of these cases are not explained by known genetic mechanisms. In addition, there are multiple reports that some patients with *BRCA1/2*-proficient OC unexpectedly also benefit from PARPi therapy (9, 14). We hypothesized that OC cells that are insensitive to PARPi therapy may display proteomic features that are primed for mediating adaptive resistance signaling, the knowledge of which could enable the development of novel biomarkers and combination therapies, a recognized continuing medical need (15). We furthermore posited that PARP1, the cognate target of PARPis, plays a central role in triggering such signaling effects, which could involve both PARP1 protein–protein interaction partners and proximal multiprotein complexes, as well as distal signaling events that are mediated by posttranslational modifications (PTMs), such as poly-ADP-ribosylation (PARylation) and phosphorylation.

Recently, we have shown that chemical proteomics with PARPis as baits is able to not just capture direct drug targets, including PARP1 and PARP2, but also stable PARP-based protein complexes that involved different DDR proteins (16). We furthermore have demonstrated that the combination of chemical proteomics with phosphoproteomics and subsequent functional validation can lead to identification of mechanistic biomarker candidates and the rational design of synergistic drug combinations (17, 18, 19).

In this study, we extended these concepts to specifically interrogate stable PARP1 protein complex partners in OC cells and determine how these proteins mount a global signaling response upon PARPi treatment. To this end, we used a comprehensive mass spectrometry (MS)–based functional proteomics approach that is comprised of chemical proteomics, phosphoproteomics, and ADP-ribosylation proteomics. Integrative analysis of these datasets indicated that OC cells that do not respond to PARPis displayed significant changes in PARP1 and PARP2 protein complex composition, as well as PTMs relevant to prosurvival AKT-mTOR signaling. Consequently, these cells exhibited prominent synergy of PARPis in combination with AKT inhibitors. In conclusion, the observation of actionable DDR and AKT/mTOR networks and subsequent validation demonstrates the potential of an unbiased integrated functional proteomics approach for discovering PARPi treatment-related signaling vulnerabilities in OC cells.

## Results

### *BRCA1*-null UWB cells are more sensitive to PARP inhibitors than *BRCA1*-reconstituted UWB+B OC cells

**Figure 1:**
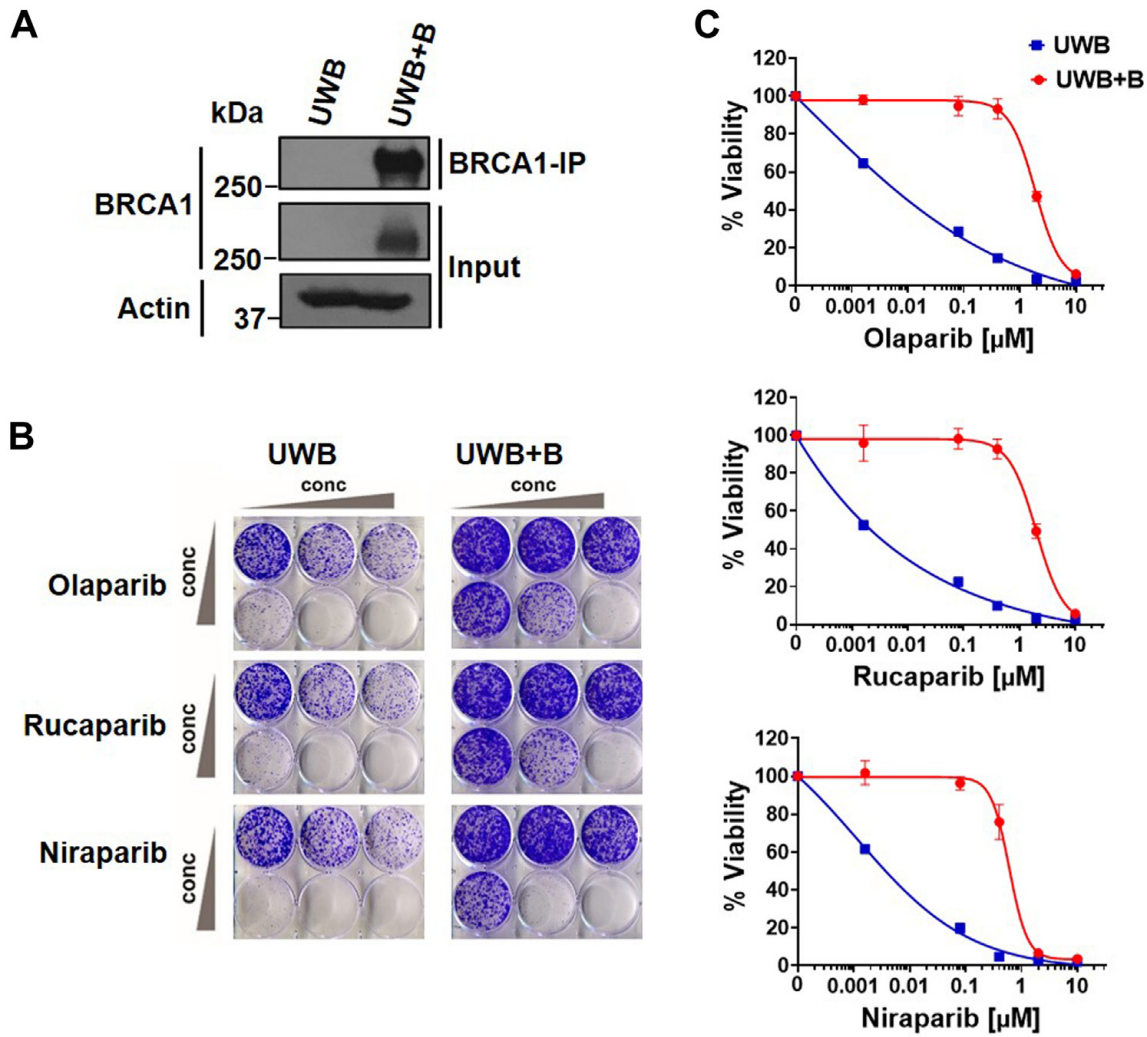
**Differential PARPi sensitivity of *BRCA1*-isogenic ovarian cancer cells.***A*, immunoprecipitation (IP) with anti-BRCA1 antibody and immunoblot analysis showing expression levels of BRCA1 in UWB and UWB+B cells. Blots are representative of two independent experiments. *B*, representative crystal violet clonogenic survival assay for UWB (3000 cells/well) and UWB+B cells (2500 cells/well) treated with olaparib, rucaparib, or niraparib at the following concentrations 0, 0.016, 0.08, 0.4, 2, 10 μM at day 1 and incubated for 10 days. *C*, quantification of crystal violet intensity. Bars represent the mean ± SD of four biological replicates.

To investigate the PARPi response in OC, we examined the efficacy of different PARPis in a pair of *BRCA1*-isogenic OC cells, that is, *BRCA1*-null UWB1.289 (UWB) cells and the corresponding UWB1.289 + BRCA1 (UWB+B) cells that express a restored WT BRCA1 function (Fig. 1*A*). UWB cells from a high grade serous ovarian carcinoma carry a germline *BRCA1* pathogenic variant within exon 11 and have a deletion of the WT allele, and the UWB+B cell line had previously been generated from parental UWB cells by stable transfection (20). Using these cells, cell viability and IC_50_ values were determined upon treatment with three different FDA approved PARPis, namely olaparib, rucaparib, and niraparib, by using crystal violet assays. Although it is possible that UWB+B cells were derived from a single clone and may feature some clone-specific properties, the *BRCA1*-null UWB cells were, as expected, more sensitive to all PARPis and reconstituted expression of *BRCA1* resulted in loss of cytotoxicity to single agent PARPi treatment (Fig. 1, *B* and *C*). Therefore, PARPi-sensitive UWB cells and PARPi-insensitive UWB+B cells were chosen to explore mechanism-based biomarkers of PARPi response and primary resistance and to identify new therapeutic targets for rational drug combinations.

### Chemical proteomics reveals different PARP1/2-Ku70/Ku80 protein complex compositions in PARPi-sensitive and PARPi-insensitive OC cells

**Figure 2:**
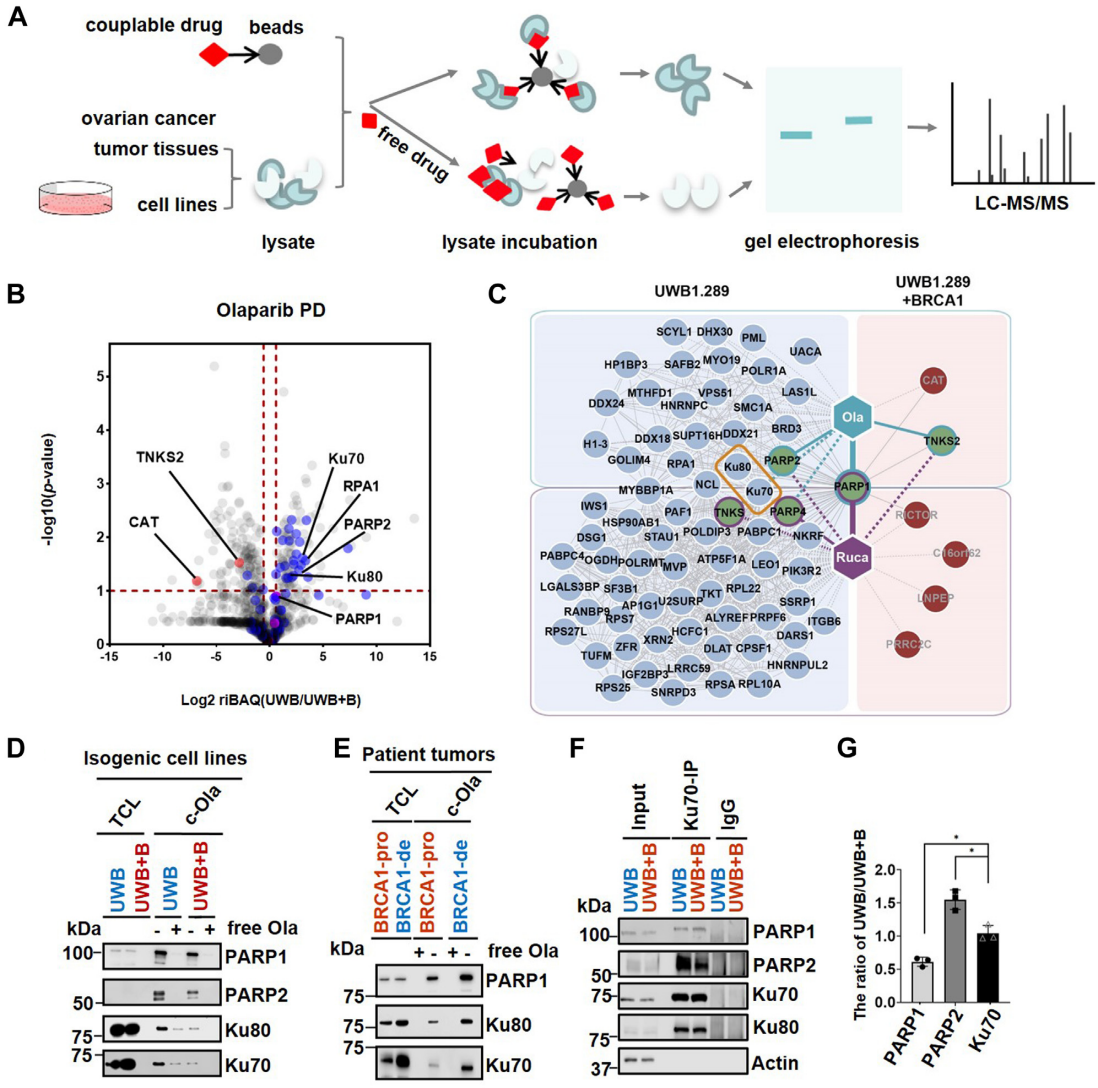
**PARPi affinity enrichment identifies differential PARP1/2 protein complexes between *BRCA1*-linked OCs.***A*, target profiling by mass spectrometry–based competitive chemical proteomics. *B*, volcano plot indicating differentially expressed interactors for the comparison of olaparib pull down (PD) between UWB and UWB+B cells. The log2 fold change of riBAQ value between two cell lines and –log10 *p* values are plotted. The interaction partners of olaparib that pass the criteria (log2 fold change of PD/CT > 1.5 and *p* < 0.05) are shown in *blue* (UWB), *red* (UWB+B), or *purple* (UWB and UWB+B). CT: competition. *C*, chemical proteomics with olaparib (top) and rucaparib (bottom) probes reveals differences in PARP1 protein complex composition between UWB (left) and UWB+B cells (right). The interaction partners that pass the criteria (log2 fold change of PD/CT > 1.5, *p* < 0.05 and ratio of riBAQ value between UWB and UWB+B > 1.5, *p* < 0.1) are depicted. The proteins in the middle of the two boxes were observed in both olaparib and rucaparib pull downs. Known direct PARPi targets are shown in *green* circles. Circle outline color denotes the drug probe used (olaparib, *green*; rucaparib, *purple*). Dashed line indicates interaction that is known but was not detected. PARP1 binder Ku70/Ku80 complex is shown in *yellow* rectangle. *D*, immunoblot of eluates from c-olaparib beads incubated with UWB or UWB+B cell lysate ±20 μM of free olaparib. Blot is representative of three independent experiments. TCL: total cell lysate. Ola: Olaparib. *E*, immunoblot of c-olaparib-modified beads incubated with lysates from frozen *BRCA1*-proficient or deficient ovarian cancer patient tumor samples, collected prior to drug therapy, ±20 μM of the free olaparib; blot is representative of three independent experiments. *BRCA1*-de: *BRCA1*-deficient ovarian cancer patient tumor samples. *BRCA1*-pro: *BRCA1*-proficient. *F*, Western blot analysis of eluates from immunoprecipitation with IgG or Ku70 antibodies. Endogenous proteins were pulled down and blotted using indicated antibodies. 20 μg of total cell lysates was loaded as the input. The Western blot image is representative of three biological replicates. *G*, densitometric analysis of Ku70 immunoprecipitation from (*F*). Relative quantification of PARP1/PARP2/Ku70 in UWB *versus* UWB+B. Data are represented as mean ± SD (indicated by the error bars). Each dot represents a biological replicate. ∗*p* < 0.05 as determined by Student’s *t* test.

We hypothesized that OC cells that do not respond to PARPi treatment display significant changes in PARP1 multiprotein complexes. To test this hypothesis, we employed a MS-based, chemical proteomics approach with PARPis as affinity baits. We have previously reported that this approach can identify not only different target profiles for each PARPi but also copurify PARP1-binding proteins (*e.g.*, LIG3, XRCC5/Ku80, XRCC6/Ku70) (16). Each PARPi analog, the syntheses of which were described previously (16) and which were successfully validated by PARP1 activity and binding assays, was individually immobilized on beads. To define differential PARP1 interacting proteins, we harnessed these olaparib and rucaparib analogs for chemical proteomics experiments with the *BRCA1*-isogenic UWB cell line pair and with OC patient tumor tissues that were collected prior to drug therapy (Fig. 2*A* and Table S1). Label-free quantitative MS analysis and subsequent data filtering were done by comparison with negative control samples derived from PARPi affinity purifications in the presence of unmodified PARPis, which scavenges targets and prevents them and their interaction partners from binding to the affinity beads. This process eliminated background proteins and highlighted several known direct drug targets, such as PARP1, PARP2, PARP4, and TNKS (Fig. 2*B* and Fig. S1*A*). In addition, analysis of the PARP1 subnetwork identified known PARP1-binding partners, such as PARP2 (which is also a direct drug target), Ku70, and NCL with both olaparib and rucaparib from UWB cells but not UWB+B cells (Fig. 2*C*). Subsequent immunoblotting of PARP1-engaged protein complex partners further confirmed higher levels of coenrichment of Ku70/Ku80 in olaparib and rucaparib drug pull downs from UWB compared with UWB+B cell lysates (Figs. 2*D* and S1*B*). Consistently, c-olaparib and c-rucaparib pull downs using frozen *BRCA1*-deficient OC tumor samples in comparison with *BRCA1*-proficient OC tumor samples, also showed increased enrichment of Ku70/Ku80 in *BRCA1*-deficient samples (Figs. 2*E* and S1*C*). Thus, these or similar PARP1:Ku70/Ku80 or PARP2 protein complex differences may determine sensitivity or primary resistance to PARPi in OC. Therefore, we examined the interaction of Ku70 with endogenous PARP1 and PARP2. Consistent with previous studies (21), Ku70 coimmunoprecipitated endogenous Ku80, PARP1, and PARP2 irrespective of *BRCA1* status. However, we observed more pronounced Ku70:PARP2 interaction in UWB cells compared with UWB+B cells. Conversely, Ku70 interaction with PARP1 was more pronounced in UWB+B than in UWB cells (Fig. 2*F* and *G*). Taken together, chemical proteomics revealed differential PARP1 and PARP2 protein complex compositions with Ku70/Ku80 between PARPi-sensitive and PARPi-insensitive OC cells.

### Global phosphoproteomics reveals DDR and EGFR signaling differences between PARPi-sensitive and PARPi-insensitive OC cells

**Figure 3:**
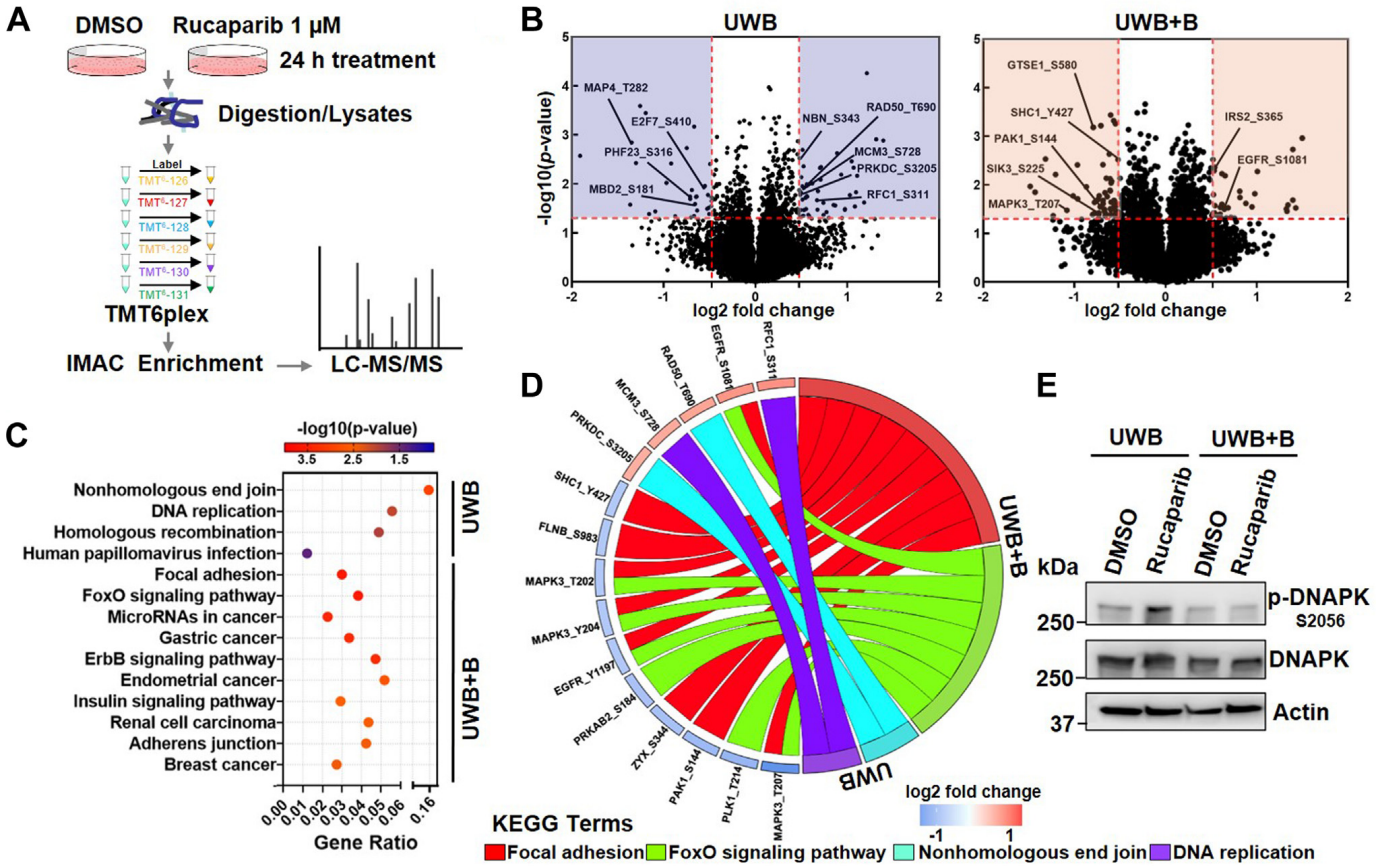
**Global phosphoproteomics analysis of network-wide signaling effects of rucaparib in *BRCA1*-isogenic OC cells.***A*, schematic of quantitative global phosphoproteomics using TMT 6-plex technology. *B*, volcano plots indicating differential protein phosphorylation in UWB (left) and UWB+B cells (right) upon treatment with rucaparib (1 μM) for 24 h compared with DMSO. Log2 fold change relative to vehicle control and –log10 *p* values were plotted. Differentially modulated phosphorylation sites that pass the criteria (absolute value of log2 fold change > 2 × average SD, *p* value < 0.05, mass error within ± 3 ppm) are shown in *blue* or *red* shaded areas. *C*, KEGG pathway analysis of phosphoproteomics data from rucaparib treatments for 24 h using Enrichr (*p* value < 0.05). *D*, chord plot of the top-2 ranked KEGG pathways of (*C*) and associated signaling changes in UWB and UWB+B cells. Individual proteins and significantly modulated phosphorylation sites are depicted. *E*, immunoblot analysis for p-DNAPK of UWB and UWB+B cells upon rucaparib treatment (1 μM, 24 h). Blots are representative of three independent experiments. DMSO, dimethyl sulfoxide.

To identify specific signaling mechanisms that are differentially affected by PARPi treatment between PARPi-sensitive and PARPi-insensitive cells, global phosphoproteomics was performed using tandem mass tag (TMT) MS-based relative quantification (Fig. 3*A*). UWB and UWB+B cells were treated with rucaparib (1 μM) or mock treated (dimethyl sulfoxide [DMSO]) for 24 h. Global enrichment of phosphopeptides was done by immobilized metal ion affinity chromatography (IMAC). After data filtering and normalization, TMT-based quantification identified 9670 pSTY phosphopeptides in UWB cells and 9929 in UWB+B cells (Table S2), of which 92 and 103 phosphopeptides, respectively, were significantly regulated by rucaparib treatment (Fig. 3*B*). KEGG pathway analysis of UWB cells revealed significant enrichment of DDR-related signaling pathways, including nonhomologous end joining (NHEJ), DNA replication, and homologous recombination. In contrast, focal adhesion, FoxO signaling, and ErbB signaling pathways were significantly enriched in UWB+B cells (Fig. 3*C*). Detailed analysis of the top 2 KEGG pathway components highlighted an increase of phosphorylation of RFC1, RAD50, MCM3, and DNAPK (PRKDC) by rucaparib in UWB cells (Fig. 3*D* and Fig. S2). In UWB+B cells, this analysis indicated a downregulation of activating phosphorylation of PAK1, MAPK3, and SHC1 in the EGFR and ErbB pathways by rucaparib, suggesting reduced signaling output in these cells by EGFR, which in turn displayed decreased phosphorylation of Y1197 and increased phosphorylation of the inhibitory serine S1081 (Figs. 3*D* and Fig. S2).

Given that chemical proteomics revealed different PARP protein complexes with Ku70/Ku80, which are important for activation of DNAPK, and that phosphoproteomics indicated increased DNAPK phosphorylation upon rucaparib in UWB cells, understanding DNAPK activity in response to rucaparib in UWB cells could be critical in predicting potential mechanisms of sensitivity. Therefore, UWB and UWB+B cells were subjected to 1 μM rucaparib and were analyzed for rucaparib-induced DNAPK phosphorylation at S2056, which is a known autophosphorylation site and thus indicative of DNAPK activity. Indeed, Western blot analysis showed that rucaparib increased DNAPK phosphorylation at S2056 in UWB cells, but not in UWB+B cells, suggesting that PARPis induce activation of NHEJ in *BRCA1*-deficient OC cells, which is consistent with increased error prone DNA repair and a role in mediating cell sensitivity to PARPis (22) (Fig. 3*E*). In summary, phosphoproteomics illustrated rucaparib effects on major cell cycle and NHEJ repair pathways in UWB cells but not in UWB+B cells.

### ADP-ribosylation proteomics reveals that PARylation affects cell cycle signaling in sensitive OC cells but not in insensitive OC cells

**Figure 4:**
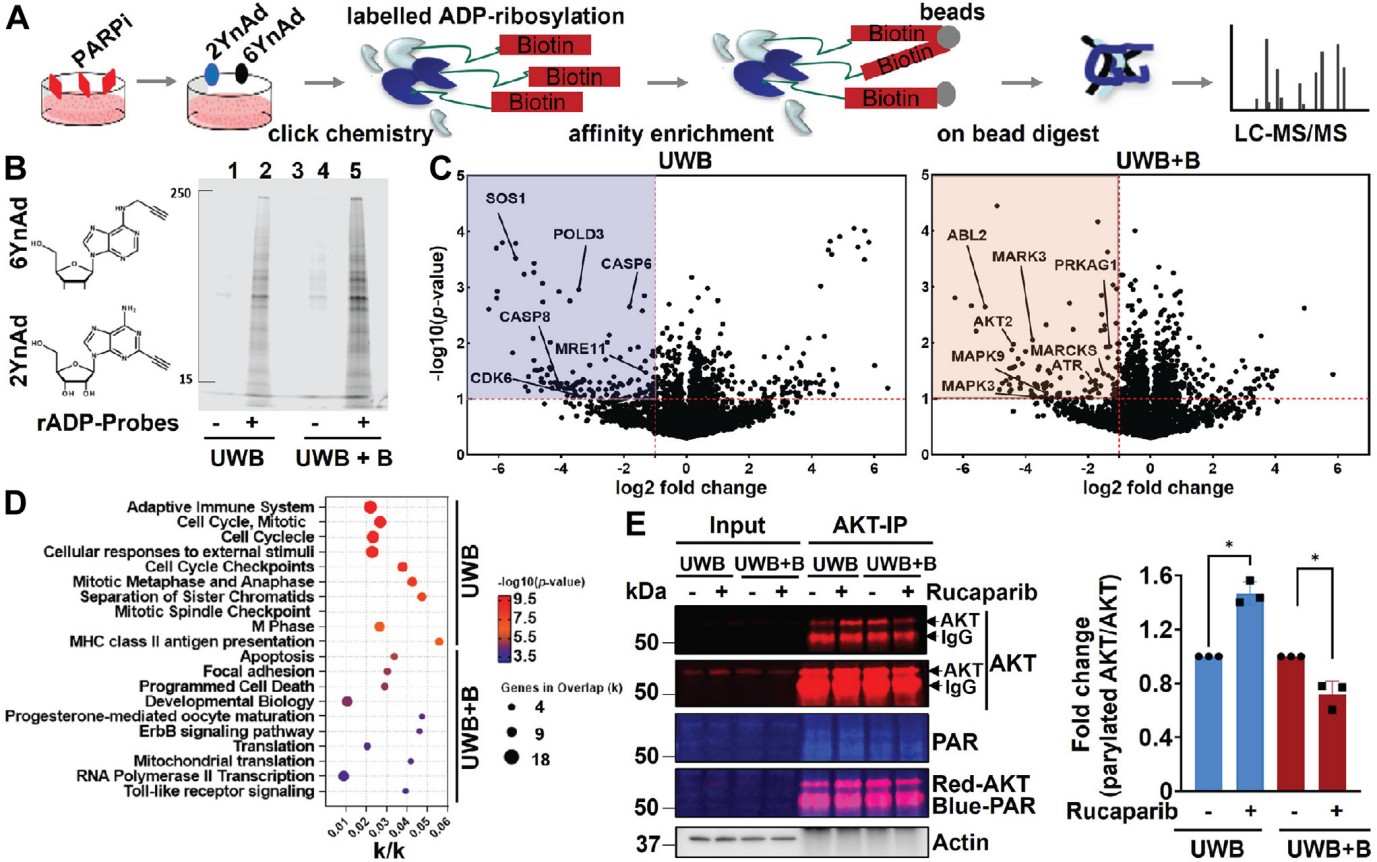
**Proteome-wide identification of ADP-ribosylation mediated signaling in response to rucaparib in *BRCA1*-isogenic OC cells.***A*, schematic illustrating the experimental workflow for identification of ADP-ribosylated proteins. UWB and UWB+B cells were treated with rucaparib (10 μM) for 1 h compared with DMSO and metabolically labeled with a mix of 6YnAd/2YnAd (0.5 mM each) for 1 h before harvest, click chemistry, affinity pull down, and label-free quantitative MS analysis. 6YnAd: 6-alkyne adenosine. 2YnAd: 2-alkyne adenosine. *B*, 6YnAd and 2YnAd probe structures and qualitative assessment of 6Yn/2YnAd labeling in UWB and UWB+B cells by in-gel fluorescence scanning. Lane 1, 4: DMSO control; lanes 2, 5: A mix of 6Yn/2Yn-adenosine (0.5 mM each); lane 3: empty. The image is representative of at least two biological replicates. *C*, volcano plots indicating differential log2 responses in the level of ADP-ribosylation of endogenous proteins to 10 μM rucaparib in UWB (left) and UWB+B cells (right). *D*, KEGG and Reactome pathway analysis of significantly modulated proteins upon rucaparib treatment. *E*, Western blot analysis of eluates from immunoprecipitation (IP) with IgG, AKT antibodies. UWB and UWB+B cells were treated with 10 μM rucaparib for 1 h and subjected to co-IP, followed by Western blotting with the indicated antibodies (left) and quantification of AKT PARylation (right). The Western blot image is representative of three biological replicates. Data are represented as mean ± SD (indicated by the error bars). Each dot represents a biological replicate. ∗*p* < 0.05 as determined by Student’s *t* test. DMSO, dimethyl sulfoxide; OC, ovarian cancer.

To identify specific PARylation-mediated signaling events differentially modulated by PARPi treatment in drug-sensitive and drug-insensitive cells, we have synthesized 6-alkyne (6Yn)- and 2-alkyne (2Yn)-adenosine probes, which have been recently reported (23, 24) to allow for specific *in situ* labeling and identification of ADP-ribosylated proteins by MS (Fig. 4*A*). We treated UWB and UWB+B cells with 0.5 mM 2YnAd and 6YnAd for 1 h in the presence of 10 μM rucaparib or DMSO. After cell lysis and copper-catalyzed azide-alkyne cycloaddition click chemistry, labeled proteins were affinity enriched using NeutrAvidin-agarose. In-gel fluorescence imaging confirmed significant enrichment of ADP-ribosylated proteins in both 2YnAd/6YnAd-treated cell lines (Fig. 4*B*). LC-MS/MS analysis identified 3431 and 3725 proteins in UWB and UWB+B cells (Table S3), respectively, of which 135 and 96 proteins were differentially ADP-ribosylated in response to rucaparib. These targets included MRE11, SOS1, GRB2, CDK6, CASP6, and CASP8 in UWB cells and AKT2, ABL2, ATR, PRKAG1, MAPK3, MAPK9, and MARCKS in UWB+B cells (Fig. 4*C*). KEGG and Reactome pathway analysis of all proteins that displayed decreased ADP-ribosylation upon rucaparib treatment in UWB cells identified a preferential enrichment for factors involved in the adaptive immune system and the cell cycle (Fig. 4*D*), several components of which were also observed by phosphoproteomics in PARPi-sensitive UWB cells, with highly significant hits such as CDC23, PSMD10, ORC1, POLD3, and MRE11. Notably, MRE11 is an important component of the MRN complex with NBN, phosphorylation of which was seen by phosphoproteomics to be significantly increased upon rucaparib treatment (Fig. 3*B*). In UWB cells, the decreased ADP-ribosylation of MRE11 and increased NBN and DNAPK activities suggest that PARPi treatment promotes MRN complex–mediated canonical NHEJ (c-NHEJ) repair. In contrast, pathway analysis of UWB+B cells suggested an enrichment of apoptosis, focal adhesion, ErbB signaling, and translation (Fig. 4*D*). Importantly, focal adhesion and ErbB signaling were also enriched in the phosphoproteomics data in PARPi-insensitive UWB+B cells. Detailed analysis of ErbB signaling pathway components highlighted decreased ADP-ribosylation of ABL2, MAPK3, MAPK9, and AKT2. Interestingly, the ADP-ribosylation of Ras/MAPK pathway associated adapter proteins SOS1 was significantly downregulated in response to rucaparib in UWB cells but not UWB+B cells.

Considering that some studies showed AKT activation to play a role in PARPi resistance in cancer (25), that DNAPK can mediate AKT activation and apoptosis inhibition in platinum resistance (26) and that AKT2 was identified only in the UWB+B cells to be ADP-ribosylated, we next evaluated AKT PARylation upon rucaparib treatment. Consistent with the proteomics data, AKT immunoprecipitation and Western blotting for PAR upon treatment of UWB and UWB+B cells with rucaparib (1 μM) for 24 h confirmed that AKT was PARylated and that AKT PARylation was significantly reduced by rucaparib treatment in UWB+B cells only (Fig. 4*E*). In summary, these data suggest that the global ADP-ribosylation patterns are profoundly different between PARPi-sensitive and PARPi-insensitive OC cells and that AKT PARylation is downregulated by PARPis specifically in PARPi-insensitive OC cells.

### Drugs that target AKT display synergy with PARPi

**Figure 5:**
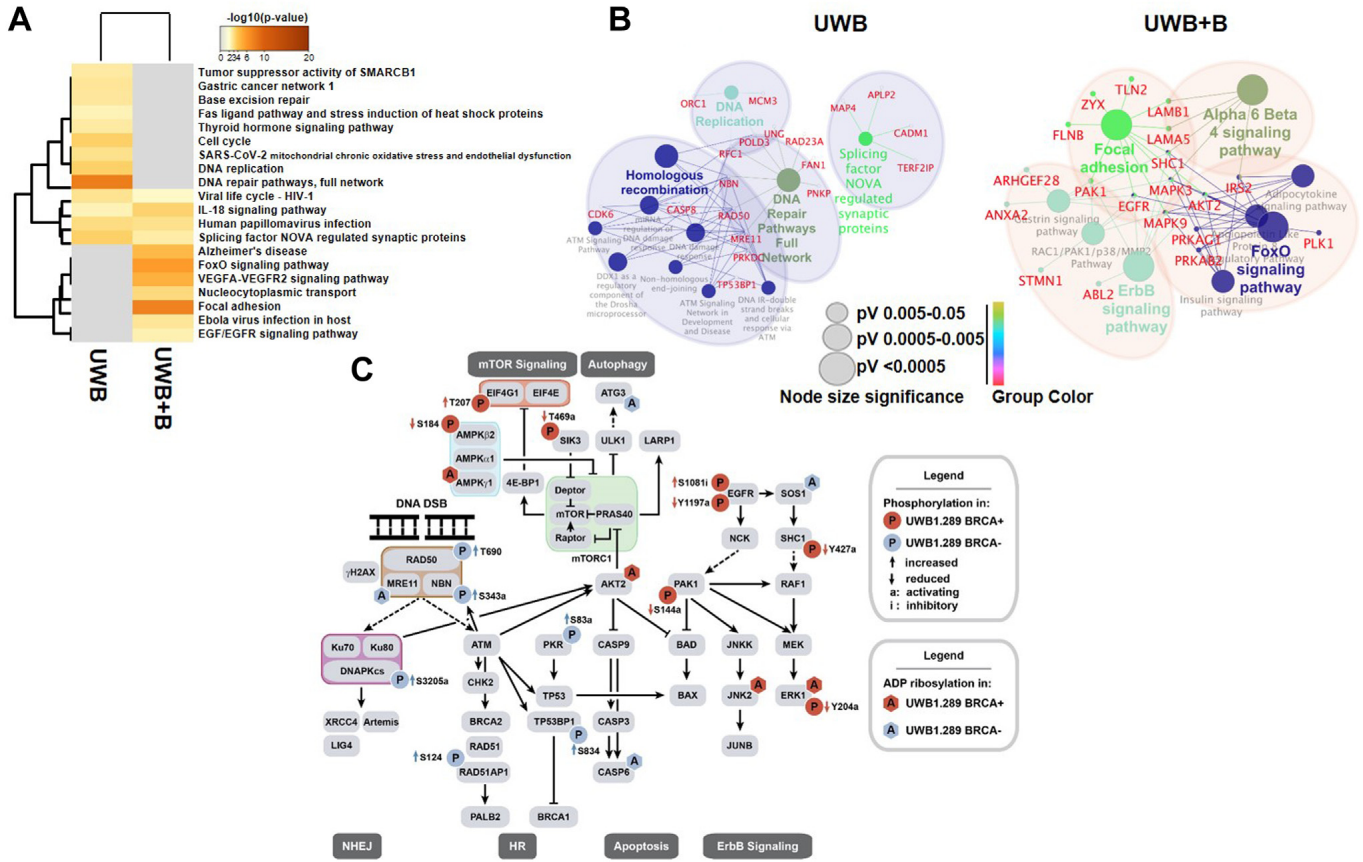
**Integrated network-based analysis indicates differential signaling responses to PARPis.***A*, heatmap showing differentially enriched clusters between UWB and UWB+B cells with modulated proteins from phosphoproteomics and ADP-ribosylation proteomics datasets in response to rucaparib combined *via* Metascape (KEGG and Wiki pathways). Setup min overlap: 3, *p* value cutoff: 0.01, min enrichment: 1.5. *Gray* color indicates lack of significance. *B*, functionally grouped network visualization using the ClueGO cytoscape plugin. The differentially significantly modulated proteins were mapped to KEGG and Wiki pathways. Node sizes indicate *p* values ≤ 0.05. Node colors indicate specific functional classes and represent various molecular pathways. *C*, integrated analysis of selected pathways and signaling changes in both cell lines. *Blue*: UWB. *Red*: UWB+B cells. Significantly modulated phosphosites (*circles*) and ADP ribosylated proteins (*hexagons*) are depicted.

To further understand the network-wide signaling effects in response to PARPis between sensitive UWB and insensitive UWB+B OC cells and considering that both phosphorylation and ADP-ribosylation data converged on similar pathways, we next integrated both datasets by combining the identified phosphoproteins and ADP-ribosylated proteins and jointly querying KEGG and Wiki pathway databases (Fig. 5, *A* and *B*). This analysis consistently enriched more DDR-related and cell cycle–related signaling pathways in UWB cells while focal adhesion, FoxO, and VEGFA-VEGFR2 signaling pathways were enriched in UWB+B cells. Detailed mapping of selected signaling changes within these pathways illustrated several ADP-ribosylation and phosphorylation events that point at differentially modulated DDR and AKT/mTORC1/AMPK signaling in these cell lines (Fig. 5*C*). Most notably, AKT appeared as a central node within the effector network in PARPi-insensitive UWB+B cells.

**Figure 6:**
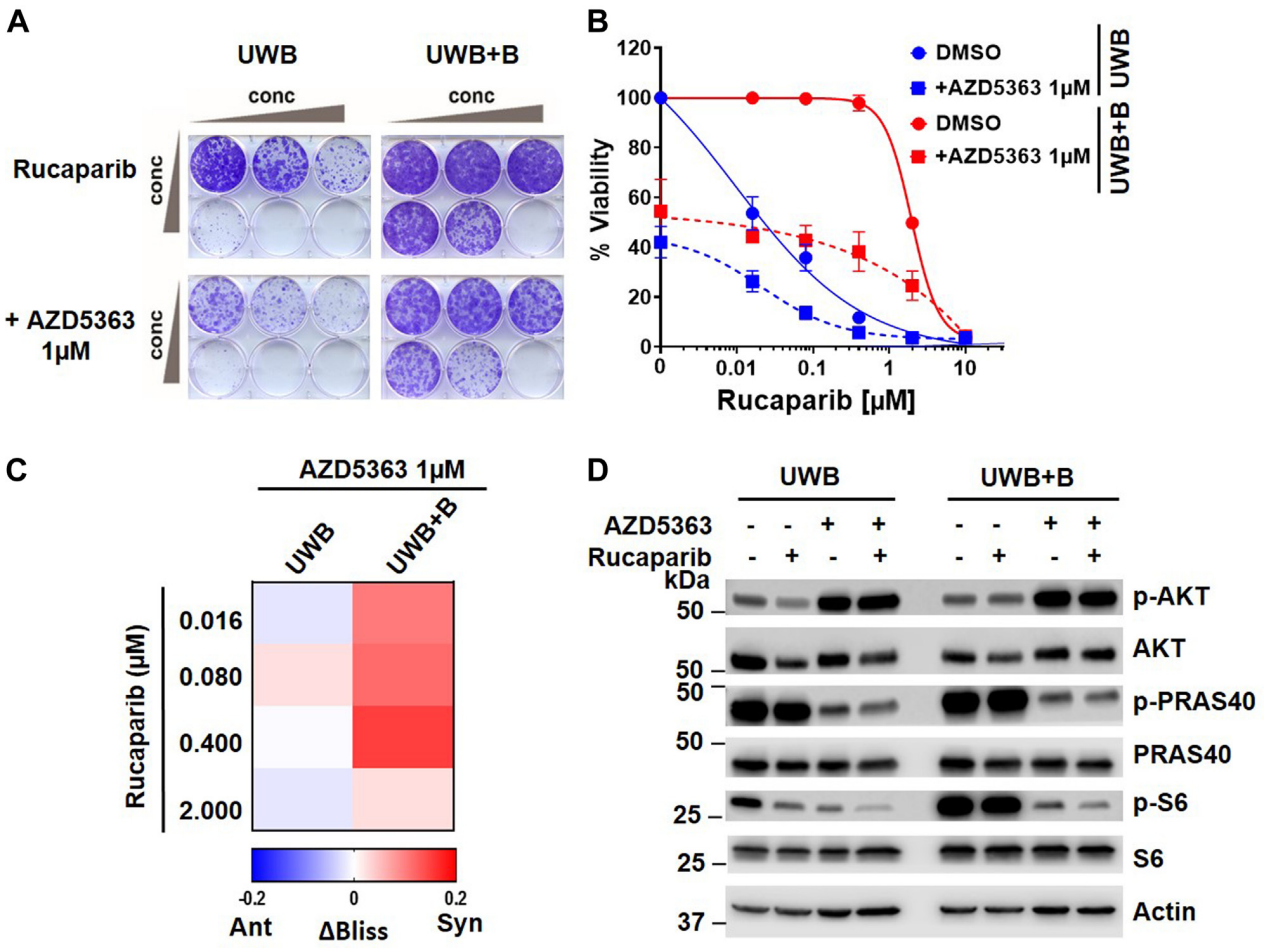
**Synergistic activity of rucaparib with AKT/DNAPK inhibitor in UWB+B cells.***A*, representative crystal violet clonogenic survival assay for UWB cells and UWB+B cells treated with rucaparib and/or the AKT inhibitor AZD5363 at the indicated concentrations at day 1 and allowed to incubate for 10 days *B*, quantification of crystal violet intensity. Error bars represent the mean ± SD of four biological replicates. *C*, synergy was determined using ΔBliss. Positive ΔBliss values indicate synergy, negative ΔBliss values antagonism. *D*, Western blot analysis of UWB and UWB+B cells upon 1 μM rucaparib treatment alone or in combination with 1 μM AZD5363 (24 h). The Western blot image is representative of three biological replicates.

We next asked, if targeting the AKT pathway enhances the efficacy of PARPis in drug-insensitive OC cells. Combination of rucaparib with the selective AKT inhibitor AZD5363 and subsequent assessment by the Bliss model of independence indicated stronger synergy across a broad concentration range in UWB+B cells compared with UWB cells (Fig. 6, *A–C*). This result was particularly pronounced for clinically relevant concentration ranges of rucaparib (∼1–2 μM) as UWB cells showed much greater sensitivity to rucaparib at these concentrations than UWB+B cells. Consistently, immunoblot interrogation of the AKT and DNAPK pathways in UWB and UWB+B cells upon treatment with rucaparib (1 μM) and/or AZD5363 (1 μM) for 24 h indicated that, as expected, AZD5363 alone effectively inhibited phosphorylation of PRAS40 (T246) and S6 (S235/236) downstream of AKT, whereas it increased phosphorylation of AKT at S473, an mTORC2 target site, in both cell lines. Notably, AKT and S6 phosphorylation were markedly decreased by rucaparib in UWB cells, whereas no significant change was apparent in rucaparib-treated UWB+B cells. However, addition of AZD5363 strongly inhibited PRAS40 and S6 phosphorylation in both cell lines. Interestingly, inhibition of AKT reduced rucaparib-induced DNAPK phosphorylation at S2056 in UWB cells (not shown), whereas in rucaparib-treated UWB+B cells, addition of AZD5363 increased DNAPK activity, which may also be indicative of the synergy between AKT inhibitors and rucaparib in these cells. Taken together, our data suggest AKT-mTORC1 signaling as a major mechanism of PARPi primary resistance in OC cells and that AKT pathway inhibition and c-NHEJ pathway activation by AZD5363 elicits strong synergistic activity with rucaparib, particularly in UWB+B cells.

## Discussion

Genomic characterization has revolutionized cancer therapy. Mutations such as *BRAF*^*V600E*^, *EGFR*^*L858R*^, or *KRAS*^*G12C*^ serve both as powerful biomarkers in melanoma and lung cancer as well as highly actionable drug targets for small molecule targeted therapeutics. In OC, loss-of-function variants in *BRCA1* and *BRCA2* have been firmly established and approved by the FDA as biomarkers that indicate defects in HRR, which causes synthetic lethality with PARPi therapy. However, only about half the patients with *BRCA*-deficient OC experience objective tumor responses upon PARPi therapy and most of such primary PARPi resistance is not explained by other genetic mutations like *BRCA* reversions (13). At the same time, clinical studies have established that not only *BRCA*-deficient but also a proportion of *BRCA*-proficient OC can respond to PARPis. Although HRR signatures or the levels and patterns of ADP-ribosylation may correlate with PARPi responses and clinical outcomes in these OCs (27), the roles and underlying mechanisms of these potential biomarkers are still incompletely understood. The DDR and DNA repair are highly dynamic and interconnected processes that involve a multitude of proteins and signaling pathways. We therefore applied an integrated MS-based functional proteomics approach including chemical proteomics, phosphoproteomics, and ADP-ribosylation proteomics to characterize PARP1 protein complexes across *BRCA1*-deficient and *BRCA1*-proficient OC cell lines and patient tumor specimens and to determine the proteome-wide effects of PARPis on OC cell signaling pathways.

The most common mechanisms of PARPi sensitivity of a *BRCA1/2*-deficient tumor are through synthetic lethality and PARP trapping (2, 28), but others include the enhancement of c-NHEJ and the inhibition of alternative NHEJ (22, 29, 30). In this study, we similarly found that rucaparib mainly affected DDR signaling in processes such as cell cycle and DNA repair pathways. This is consistent with the reported mechanisms of PARP inhibitors. Thus, synthetic lethality with PARPi in UWB cells could be reflected by the accumulation of unrecognized DNA damage and NHEJ repair. In addition, our findings provide insight into specific signaling events. Consistently, we observed that rucaparib treatment induces ATM-dependent autophosphorylation of DNAPK, a key mediator to facilitate c-NHEJ in *BRCA1*-deficient, PARPi-sensitive UWB cells. It has been reported that PARP1 inhibits the c-NHEJ repair by preventing the binding of the Ku70/Ku80 proteins to free DNA ends. Once PARP1 is loaded to the dsDNA breaks, it will remove the Ku complex *via* PARylation (31). Consistent with this finding, we observed that PARP/Ku70/Ku80 complexes are highly enriched by PARPi pull down in PARPi-sensitive UWB cells compared to PARPi-insensitive UWB+B cells. Although Ku80 interacts with BRCA1, which could compete for Ku80 in *BRCA1*-proficient cells, this is unlikely to account for the difference because Ku80 interaction with BRCA1 is lower in stages of the cell cycle in which levels of BRCA1 are the highest (S/G2), suggesting cell cycle–specific PTMs may modulate the interaction (32). Interestingly though, we observed that much of this difference can be attributed to binding of Ku70/Ku80 to PARP2 rather than PARP1. Our results suggest that PARPis prevent Ku70/Ku80 PARylation by PARP1/2 and removal of the Ku complex from DNA lesions thereby promoting DNAPK activity-mediated c-NHEJ repair. This hyperactivation of c-NHEJ increases the likelihood of catastrophic genomic instability and subsequent cell death. A recent MS study identified VCP in the PARP1 WT breast cancer cells as a PARP1-associated protein and suggested that VCP plays a key role in the processing of trapped PARP1 (33). In our study in OC cells, we detected VCP as a PARPi-interacting protein but observed no statistically significant difference between PARPi-sensitive UWB cells and PARPi-resistant UWB+B cells.

Notably, PARP inhibition has also been reported to activate AKT resulting in cyto-protective and mitochondria-protective actions in oxidative stress (34). AKT is a kinase that controls physiological processes such as cell growth, proliferation, the cell cycle, and glycometabolism (35, 36, 37, 38). Dysregulation of the AKT pathway is well described in cancer and has been implicated in tumorigenesis and resistance to chemotherapy. Here, we found that AKT-mTORC1 survival signaling was more downregulated by rucaparib in UWB cells compared with UWB+B cells. In addition, we observed that AKT was PARylated and that AKT PARylation was reduced by rucaparib treatment in UWB+B cells but not UWB cells. As we also detected relatively high basal AKT-mTORC1 prosurvival signaling, as indicated by higher pPRAS40, and particularly pS6 levels, in UWB+B cells compared to UWB cells, AKT PARylation may help maintain signaling output from this pathway. Notably, combined inhibition of PARP1 and AKT, which has been described as a potential therapeutic modality in *BRCA1/2*-deficient cells (39) showed a strong synergistic effect in UWB+B cells, particularly at physiologically relevant rucaparib concentrations. Our findings are consistent with a recent report describing efficacy of combined PARP1 and AKT targeting also in *BRCA1*-proficient OC (40).

Through integration of multiproteomics, our data suggest that PARPis engaged differential PARP1/PARP2 and Ku70/Ku80 complexes thereby mediating NHEJ, the hyperactivity of which is mainly responsible for the lethality in PARPi-sensitive UWB cells. In addition, AKT PARylation and increased AKT-mTORC1 signaling are associated with PARPi insensitivity in UWB+B cells. We therefore believe that the degree of association of PARP1/PARP2 with Ku70/Ku80 complexes and AKT PARylation constitute novel biomarker candidates that also mechanistically contribute to PARPi sensitivity for OCs and could be determined by immunohistochemistry. In addition, the new insights into the association of AKT with ADP-ribosylation provide a compelling molecular basis for developing PARP and AKT inhibitor combinations. At the same time, positive results of a phase I trial (41) and an ongoing clinical trial combining PARPi and AKT inhibitors further confirmed the translational therapeutic regimen to extend the therapeutic potential of PARP inhibitors for OC. Moreover, we believe this novel integrated proteomics approach is a valuable and powerful tool that provides a complementary and more comprehensive understanding of PARPi primary resistance and offers an opportunity to expedite translation of basic research to more precise treatment in the clinic.

## Experimental procedures

### Cell lines and cell culture

UWB1.289 (UWB) and UWB1.289 + BRCA1 (UWB+B) cell lines were purchased from American Type Culture Collection (ATCC). Cells were incubated in 1:1 MEBM Bullet Kit and RPMI1640 media (Lonza) supplemented with 3% fetal bovine serum and maintained with 5% CO_2_ at 37 °C. UWB+B cells were furthermore supplemented with 200 μg/ml G418 (Life Technologies, Inc). Cells were periodically tested for *mycoplasma* and authenticated using Short Tandem Repeat analysis.

### Patient samples

Deidentified OC patient tumor samples were obtained from the Tissue Core Facility at the Moffitt Cancer Center and were collected from two chemotherapy-naive patients who had undergone surgery, snap frozen in liquid nitrogen, and stored at −80 °C. Written informed consent was obtained in accordance with the Declaration of Helsinki and the study was deemed by Moffitt Scientific Protocol Review & Monitoring System Committee as Non-human subject research (MCC 50307). Patients were consented to the Total Cancer Care Protocol, the Moffitt Cancer Center's institutional biorepository (MCC#14690; Advarra IRB Pro00014441). TNM stage IV ovarian tumor (ICD C56.9) samples with a serous cystadenocarcinoma histology were derived from patients with <60 years of age.

### Compounds

Olaparib (AZD2281; Chemietek), niraparib (MK4827; Chemietek), rucaparib (AG014699; Chemietek), and AZD5363 (Active Biochem) were dissolved in DMSO at a concentration of 10 mM and stored at −20 °C. 6-alkyne (6Yn)- and 2-alkyne (2Yn)-adenosine probes were synthesized in-house by the chemistry unit of the Chemical Biology Core of the Moffitt Cancer Center. Drug dilutions were made in DMSO.

### Immunoblotting

Cells were harvested from culture plates, washed three times with ice-cold PBS, and lysed in Laemmli buffer containing 0.4% NP40 alternative. The lysates were precleared twice by centrifugation at 27,000*g* at 4 °C for 20 min and protein concentration was determined using standard Bradford assay. Proteins were resolved by SDS-PAGE, transferred to activated polyvinylidene difluoride membranes using the TransBlot Turbo system (Bio-Rad), incubated at 4 °C overnight with primary antibodies, and washed with Tris-buffered saline with Tween-20, followed by 1 h incubation with secondary antibodies at room temperature (RT). Signals were developed with Clarity Western ECL Substrate (Bio-Rad, 1705061) and read on an Odyssey FC Imager using Image studio software (Licor). Antibodies used were against BRCA1 (#OP92, Sigma–Aldrich), AKT (#9272S, Cell Signaling), phospho-AKT (pSer473) (#9271S, Cell Signaling), PRAS40 (#2691S, Cell Signaling), phospho-PRAS40 (pThr246) (#13175S, Cell Signaling), S6 Ribosomal (#2217S, Cell Signaling), phospho-S6 (pSer235/236) (#4858S, Cell Signaling), PAR (#4335-MC-100, Trevigen), and actin (#A5441,Sigma). Secondary antibodies were horseradish peroxidase–conjugated α-rabbit or α-mouse (GE Healthcare), IRDye 800CW goat anti-rabbit IgG secondary antibody(92632211, LI-COR Biosciences), and IRDye 680RD goat antimouse IgG secondary antibody(92,668,070, LI-COR Biosciences).

### Crystal violet cell viability assay

Cells were plated in a 6-well plate at 2500 cells/well and 3000 cells/well for UWB+B and UWB cells, respectively, and treated with the appropriate drugs for a total of 10 days. Cells were fixed with methanol and stained with 0.1% crystal violet solution before imaging using a tabletop scanner. Crystal violet was quantified using methanol extraction and analyzed at 540 nm on a M5 Spectramax plate reader (Molecular Devices). Data were normalized to vehicle-treated wells and fit to a sigmoidal dose-response curve using GraphPad Prism 9 software (GraphPad Software Inc). Drug combination effects were assessed by the Bliss independence model (42). If the combined effect was greater than expected for each drug additively, the response was classified as synergistic (Bliss score >0), while antagonism is concluded when the combination produces less than the expected additive effect (Bliss score <0).

### Chemical proteomics

We have previously described the synthesis and validation of c-olaparib and c-rucaparib (16). Immobilization and drug pull-down experiments were performed as previously described in a step-by-step protocol (43) with modifications as follows: c-olaparib and c-rucaparib were immobilized on NHS-activated Sepharose beads by overnight RT incubation in the presence of triethylamine. Successful coupling was confirmed using HPLC-MS and beads were blocked overnight with ethanolamine. Lysates (5 mg per sample) were preincubated with competition compound (20 μM) or DMSO for 30 min at 4 °C. The drug beads were washed with DMSO followed by lysis buffer and affinity pull-down experiments were performed by incubating drug beads with lysates for 2 h at 4 °C. Beads were further washed on Bio-spin disposable chromatography columns (Bio-Rad) with lysis buffer, bound proteins were eluted by heating to 95 °C in 30 μl of Laemmli buffer for 5 min. A portion of each eluate was set aside for analysis by Western blotting. The eluates were run on SDS-PAGE, followed by in-gel trypsin digestion, and cleared with C18 ZipTip clean up (Millipore). Briefly, the ZipTips were activated by pipetting methanol a few times followed by 50% acetonitrile in 0.1% TFA and 2% acetonitrile in 0.1% TFA. Samples were then pipetted up and down a few times and tips were washed with 2% acetonitrile in 0.1% TFA and eluted in 50% acetonitrile in 0.1% TFA. Eluates were concentrated using vacuum centrifugation. The peptides were redissolved in HPLC buffer spiked with Pierce retention time calibration mixture (Thermo). LC-MS/MS analysis using a Dionex RSLCnano UHPLC interfaced with a Q Exactive Plus mass spectrometer (Thermo) was performed as described previously (17). Data were searched against the SwissProt 2018 human protein database (downloaded 2018_05, 20,292 entries) using the MaxQuant search engine (version 1.5.2.8). Up to two missed cleavages by trypsin were allowed and carbamidomethylation of cysteine and methionine oxidation were selected as variable modifications. First, mass tolerance for intact peptides was set to 20 ppm, fragment ion tolerance was m/z ± 0.05, and main search peptide tolerance was 4.5. The data were filtered for 1% false discovery rate (FDR), plus common contaminants, reverse hits, and intensity value = 0. Missing values were imputed with the lowest value of each column and iBAQ intensities were Log2 transformed. A cutoff of 1.5 for Log2 ratios of pull downs and competition controls was applied. To compare two cell lines, the relative iBAQ (iBAQ/∑iBAQ) from pull-down samples was calculated and the fold change cutoff of riBAQ between UWB and UWB+B was set to 1.5. The experiment was performed with three biological replicates.

### Global phosphoproteomics

UWB and UWB+B cells were treated with rucaparib (1 μM) for 24 h. Cells were harvested, lysed with urea lysis buffer, sonicated using a microtip sonicator, and digested. Peptides were extracted using the Cell Signaling protocol (#8803 Cell Signaling Technology). TMT labeling, IMAC enrichment, MS analysis, and data search were performed as described earlier (44). Briefly, samples were labeled using TMT reagents as described by the manufacturer (Thermo Fisher TMT sixplex Isobaric Mass Tagging Kit, #90064). Labeling efficiency was confirmed by MS. After sample combination and lyophilization, peptides were redissolved in 250 μl of 20 mM ammonium formate buffer (pH 10.0). Basic pH reversed-phase liquid chromatography separation was performed on an XBridge column (Waters). Twelve concatenated peptide fractions were dried by vacuum. pSTY peptides were further enriched using IMAC magnetic beads (CST) with a KingFisher robot (ThermoFisher). All samples were spiked with Pierce retention time calibration mixture standard peptides to confirm consistent performance of the LC-MS analyses. The acquired LC-MS/MS data were searched with MaxQuant (pSTY) with the human UniProt database using the embedded search engine, Andromeda (45). Carbamidomethylated cysteines were set as fixed modification and oxidation of methionine, N-terminal protein acetylation, and phosphorylation of serine, threonine, and tyrosine as variable modifications. Further, the MaxQuant initial search precursor and fragment ion tolerance were set to 20 ppm and m/z 0.05, respectively. The MaxQuant main search precursor ion mass tolerance was 4.5 ppm. Resolution was set to 70,000 at 200 m/z for MS1 and 17,500 for MS2. MaxQuant automatically filters out any TMT-labeled peptides that are isolated with less than 75% purity. The data were then filtered for 1% protein FDR, plus common contaminants (*e.g*., nonhuman proteins, etc.). For the analysis and comparison of TMT 6-plex global pSTY data, the reporter ion intensity was used for the relative quantification of each peptide. IRON (iterative rank-order normalization) of MaxQuant data was performed as described before (46). The proteins with phosphorylations were filtered for absolute value of log_2_ fold change (>2 the average standard deviation) and *p* value (<0.05). The experiment was performed with three biological replicates. Each treated sample was run as two technical replicates.

### ADP-ribosylation proteomics

These experiments were performed essentially as described by Kalesh *et al*. (23). Briefly, UWB and UWB+B cells were treated with 10 μM rucaparib or DMSO for 1 h, medium was removed, and the cells were treated with 0.5 mM 6YnAd and 0.5 mM 2YnAd for an additional hour. Whole cell lysates were prepared and proteins were quantified. An aliquot of total protein (500 μg) per each condition were subjected to click reactions using Azide-PEG3-Biotin or Azide-TAMRA-Biotin capture reagent. Proteins were precipitated using the methanol/chloroform/water system and air-dried precipitates were resuspended. An aliquot of protein (30 μg) was loaded per gel lane (4%–15% mini-PROTEAN TGX precast gels, Bio-Rad) and resolved by SDS-PAGE. The gels were scanned for fluorescence labeling using a GE typhoon 5400 gel scanner and 500 μg of protein was subjected to affinity enrichment on NeutrAvidin-Agarose beads. After extensive washing, the beads were subjected to 3 mM DTT treatment, 10 mM iodoacetamide treatment, and overnight trypsinization. Samples were acidified to pH 3 using formic acid, allowed to stand for 5 min, centrifuged, and the supernatant was collected. Beads were washed with 0.1% formic acid solution in water, centrifuged, and supernatants were mixed with the previous supernatants. The collected tryptic peptides were cleared with C18 ZipTip clean up (Millipore). Peptides were processed and analyzed as described previously (see Chemical proteomics). The data were filtered using 1% FDR. Missing values were imputed with the lowest value of each column and intensities were Log2 transformed. Data analysis was done by comparison of rucaparib with DMSO control at 1 h. The fold change of intensity value was set to 2. The experiment was performed with three biological (independent) replicates.

### Quantification and statistical analysis

Data were obtained from three independent experiments and shown as the mean ± SD. Data were analyzed using Microsoft Excel and GraphPad Prism 9 software. Statistical analyses were performed using Student’s *t* test and synergy was determined by the Bliss independence model. All statistical details are included in the figure legends. All MaxQuant data were first filtered for peptides with PEP score < 0.05. Furthermore, reverse and contaminant peptides and peptides with no intensity were excluded. Data were then normalized using IRON (46). Phosphoproteomics data analysis was done by comparison of rucaparib with DMSO control at 24 h. Only phosphopeptides within a mass tolerance of ± 3 ppm were selected. All data across treatments were first normalized to DMSO-treated samples.

### Gene set enrichment analyses and data visualization

KEGG pathway enrichment analysis of differential phosphorylated genes from UWB or UWB+B cells was performed with Enrichr (https://maayanlab.cloud/Enrichr/) (47, 48, 49). ADP-ribosylated genes from UWB or UWB+B cells were analyzed separately (KEGG and Reactome gene sets, GSEA) (49, 50). Bubble Plots were created by GraphPad Prism 9. KEGG Chord was plotted with https://www.bioinformatics.com.cn/en, a free online platform for data analysis and visualization. Heatmap of enriched terms (KEGG and Wiki pathways) across input gene lists was created by Metascape, colored according to *p*-values (51). Regulated genes from phosphoproteomics and ADP-ribosylation proteomics were provided as an input to Cytoscape 3.8.2 software (https://cytoscape.org/) for KEGG terms and Wiki pathways using functional analysis modules of ClueGo and Cluepedia tools (52). Protein–protein interaction analysis was carried out with all protein interaction databases using the Metascape tool.

## Data availability

Proteomics data are available *via* ProteomeXchange at http://www.ebi.ac.uk/pride with identifiers PXD029424 (Chemical proteomics), PXD029432 (Phosphoproteomics), and PXD029428 (ADP-ribosylation proteomics).

## Supporting information

This article contains supporting information.

## Conflict of interest

J. M. K. reports support from Bristol-Myers Squibb on an unrelated project. All other authors declare that they have no conflict of interest with the contents of this article.

## Supporting information

Supplemental Table S1–S3

Supplemental Figure S1 **PARPi affinity enrichment identifies differential PARP1/2 protein complexes between *BRCA1*-linked OCs.***A*, volcano plot indicating differentially expressed interactors for the comparison of rucaparib pull down (PD) between UWB and UWB+B cells. The log2 fold change of riBAQ value between two cell lines and –log10 p values are plotted. The interaction partners of rucaparib that pass the criteria (log2 fold change of PD/CT > 1.5 and p < 0.05) are shown in *blue* (UWB), *red* (UWB+B), or *purple* (UWB and UWB+B). *B*, immunoblot of eluates from c-rucaparib beads incubated with UWB or UWB+B cell lysate ±20 μM of free rucaparib. Blot is representative of three independent experiments. TCL: total cell lysate. Ruca: rucaparib. *C*, immunoblot of c-rucaparib-modified beads incubated with lysates from frozen BRCA1-proficient or BRCA1-deficient ovarian cancer patient tumor samples, collected prior to drug therapy, ±20 μM of the free rucaparib; blot is representative of three independent experiments. BRCA1-de: BRCA1-deficient ovarian cancer patient tumor samples. BRCA1-pro: BRCA1-proficient

Supplemental Figure S2 **MS/MS spectra of the selected differential phospho-peptides depicted in**Figure 3**D**.
