# Supplementary figures and images for: Integrated proteomics identifies PARP inhibitor-induced prosurvival signaling changes as potential vulnerabilities in ovarian cancer
Source: J Biol Chem. 2022 Sep 29;298(11):102550. doi: 10.1016/j.jbc.2022.102550 (PMC9636579; doi:10.1016/j.jbc.2022.102550)

# Rucaparib PD

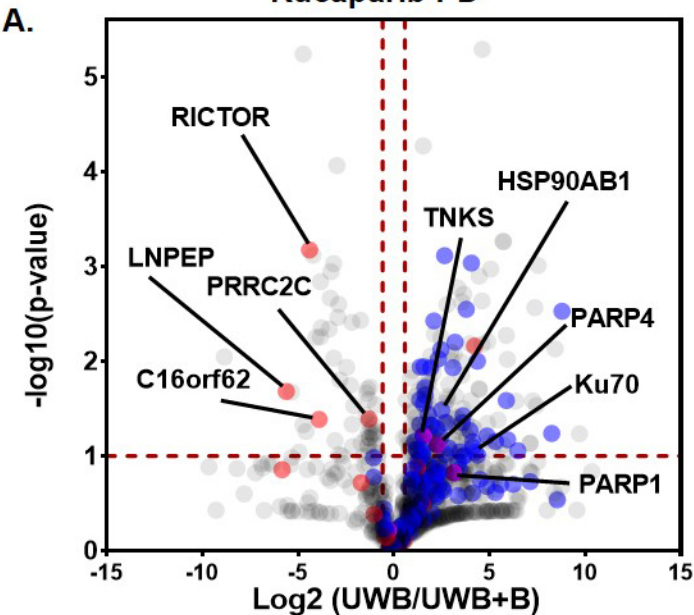

**B.**

## Isogenic cell lines

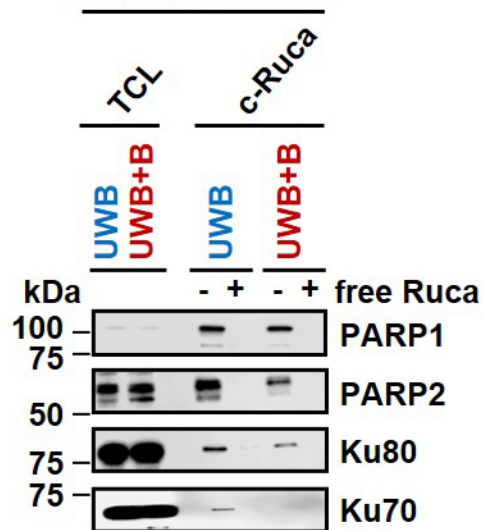

**C.**

## Patient tumors

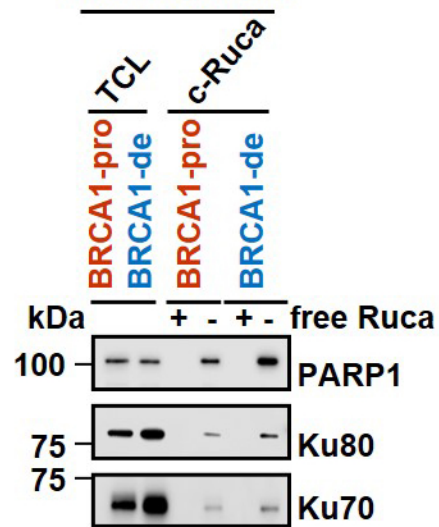

Supplement: Supplemental Figure S1 — PARPi affinity enrichment identifies differential PARP1/2 protein complexes between BRCA1-linked OCs.A, volcano plot indicating differentially expressed interactors for the comparison of rucaparib pull down (PD) between UWB and UWB+B cells. The log2 fold change of riBAQ value between two cell lines and –log10 p values are plotted. The interaction partners of rucaparib that pass the criteria (log2 fold change of PD/CT > 1.5 and p < 0.05) are shown in blue (UWB), red (UWB+B), or purple (UWB and UWB+B). B, immunoblot of eluates from c-rucaparib beads incubated with UWB or UWB+B cell lysate ±20 μM of free rucaparib. Blot is representative of three independent experiments. TCL: total cell lysate. Ruca: rucaparib. C, immunoblot of c-rucaparib-modified beads incubated with lysates from frozen BRCA1-proficient or BRCA1-deficient ovarian cancer patient tumor samples, collected prior to drug therapy, ±20 μM of the free rucaparib; blot is representative of three independent experiments. BRCA1-de: BRCA1-deficient ovarian cancer patient tumor samples. BRCA1-pro: BRCA1-proficient [file mmc2.pdf]
