# Supplementary material for: Integrated proteomics identifies PARP inhibitor-induced prosurvival signaling changes as potential vulnerabilities in ovarian cancer
Source: J Biol Chem. 2022 Sep 29;298(11):102550. doi: 10.1016/j.jbc.2022.102550 (PMC9636579; doi:10.1016/j.jbc.2022.102550)

EGFR_S1081

YSSDPTGALT(0.003)EDS(0.869)IDDT(0.128)FLPVPEYINQSVPK

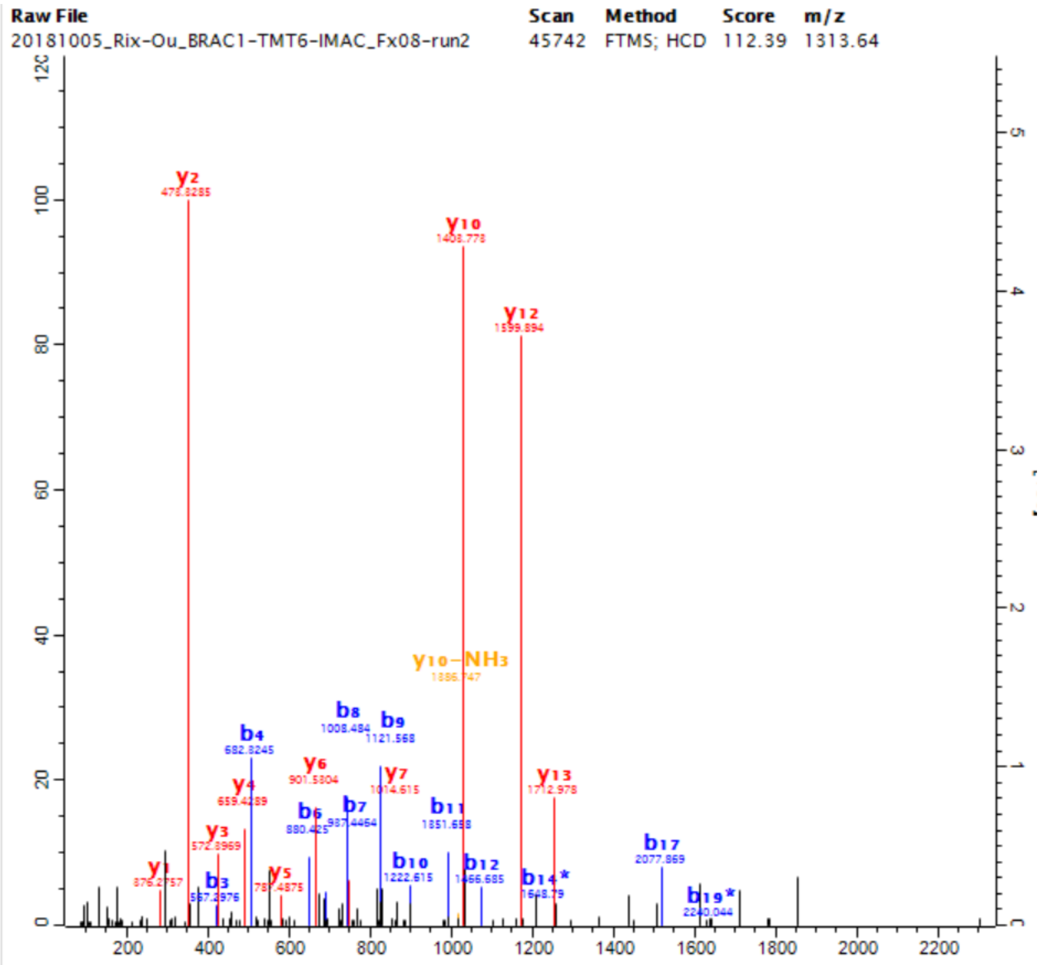

PRKDC_S3205

LTPLPEDNS(1)MNVDQDGDPSDR

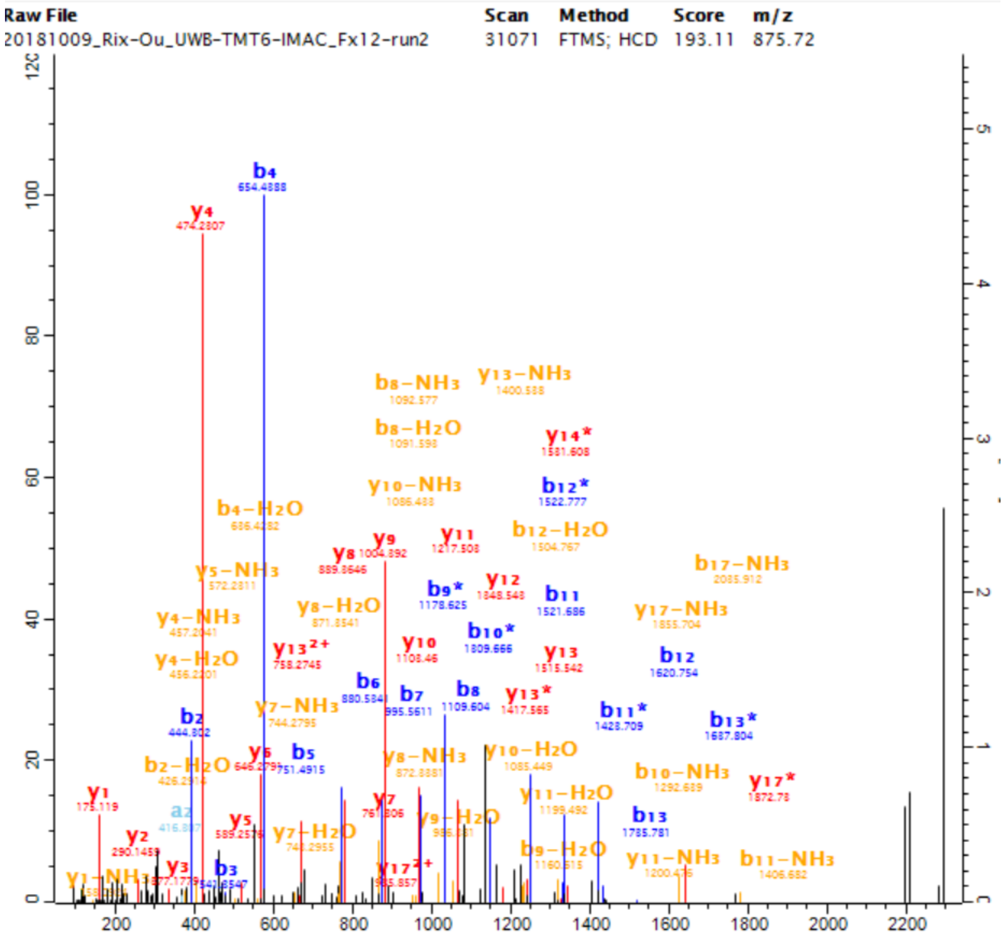

SHC1_Y427

ELFDDPS(0.001)Y(0.999)VNVQNLDK

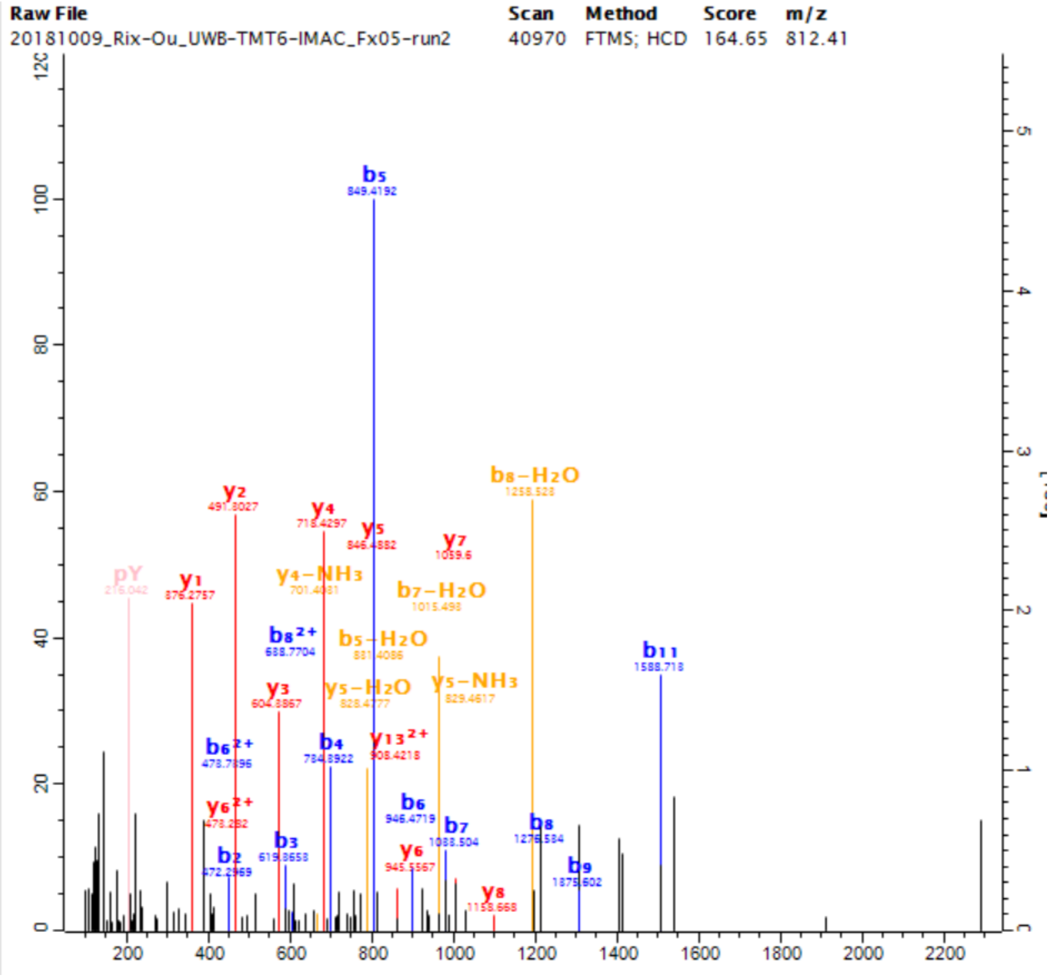

RAD50_T690

VFQT(1)EAELQEVISDLQSK

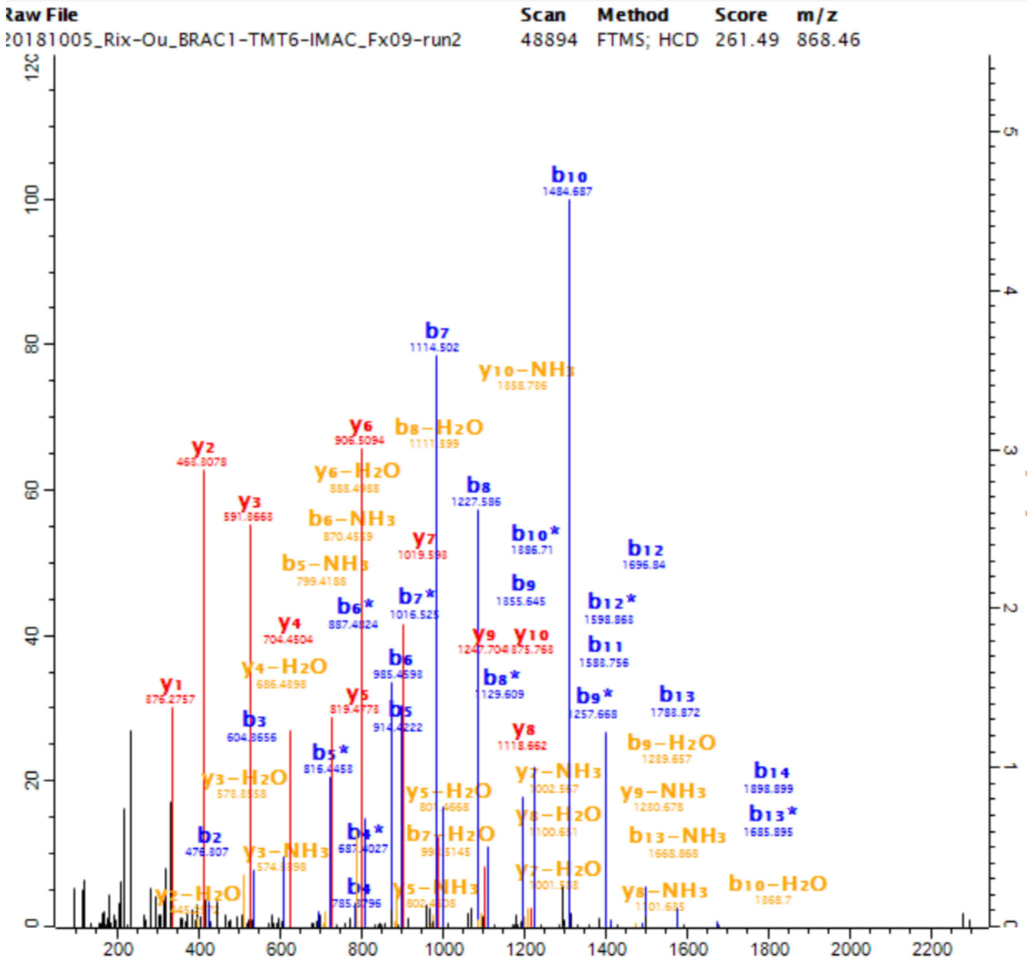

PRKAB2_S184

DLS(0.002)S(0.011)S(0.98)PPGPY(0.007)GQEMYAFR

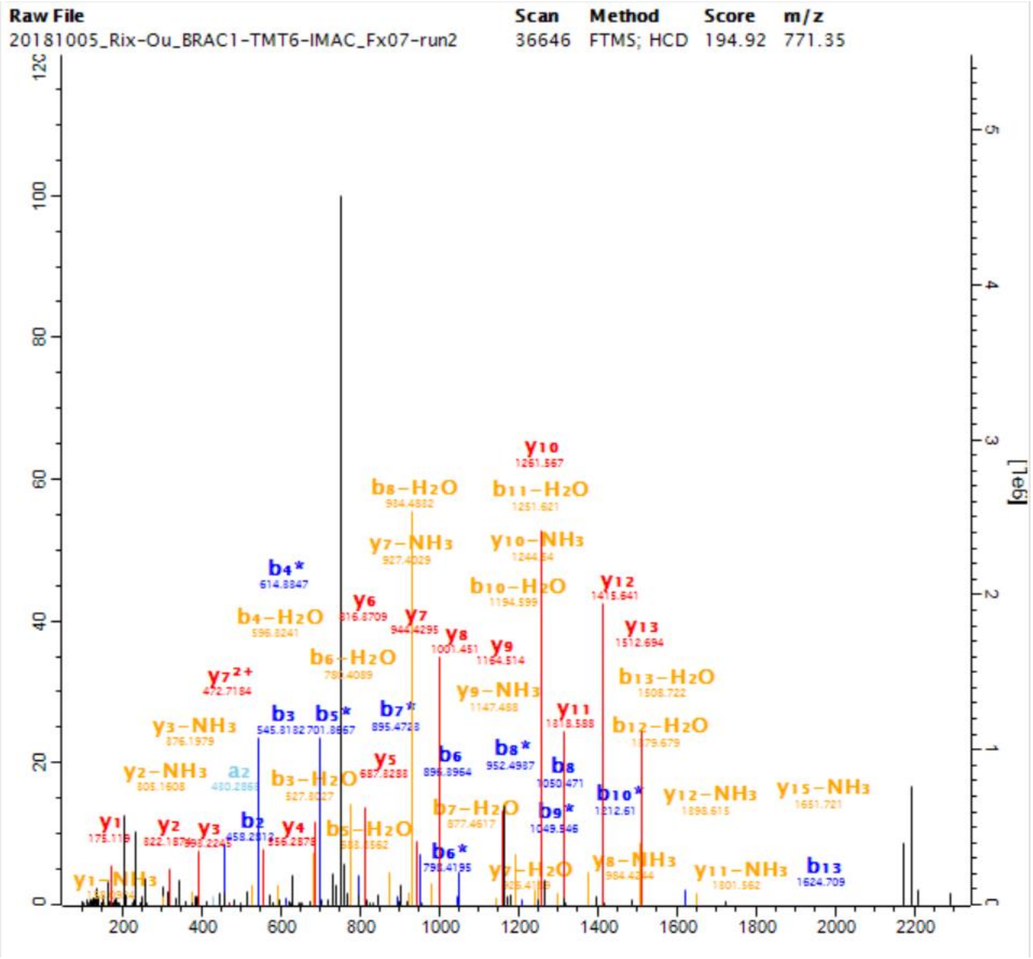

RFC1_S311

SSADKIGEVS(0.69)S(0.31)PK

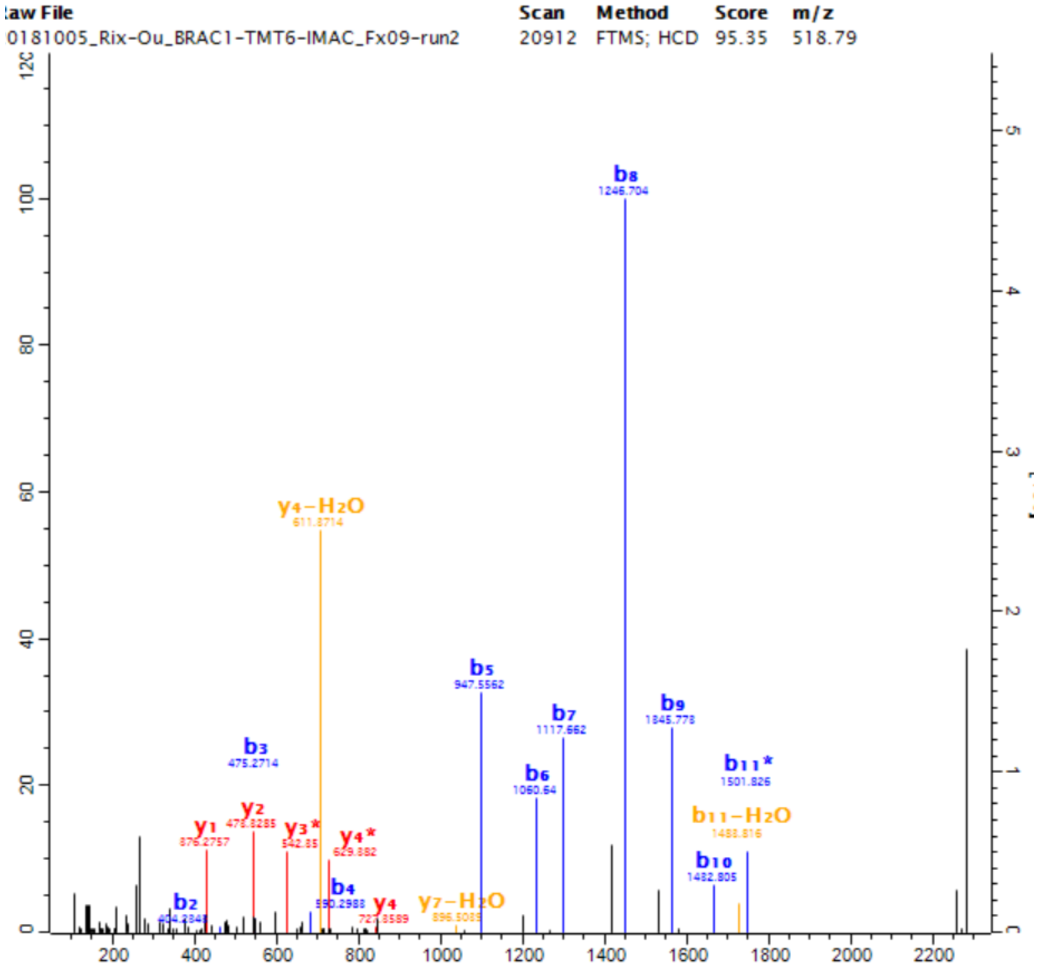

FLNB_S983

LDVT(0.001)ILS(0.997)PS(0.003)R

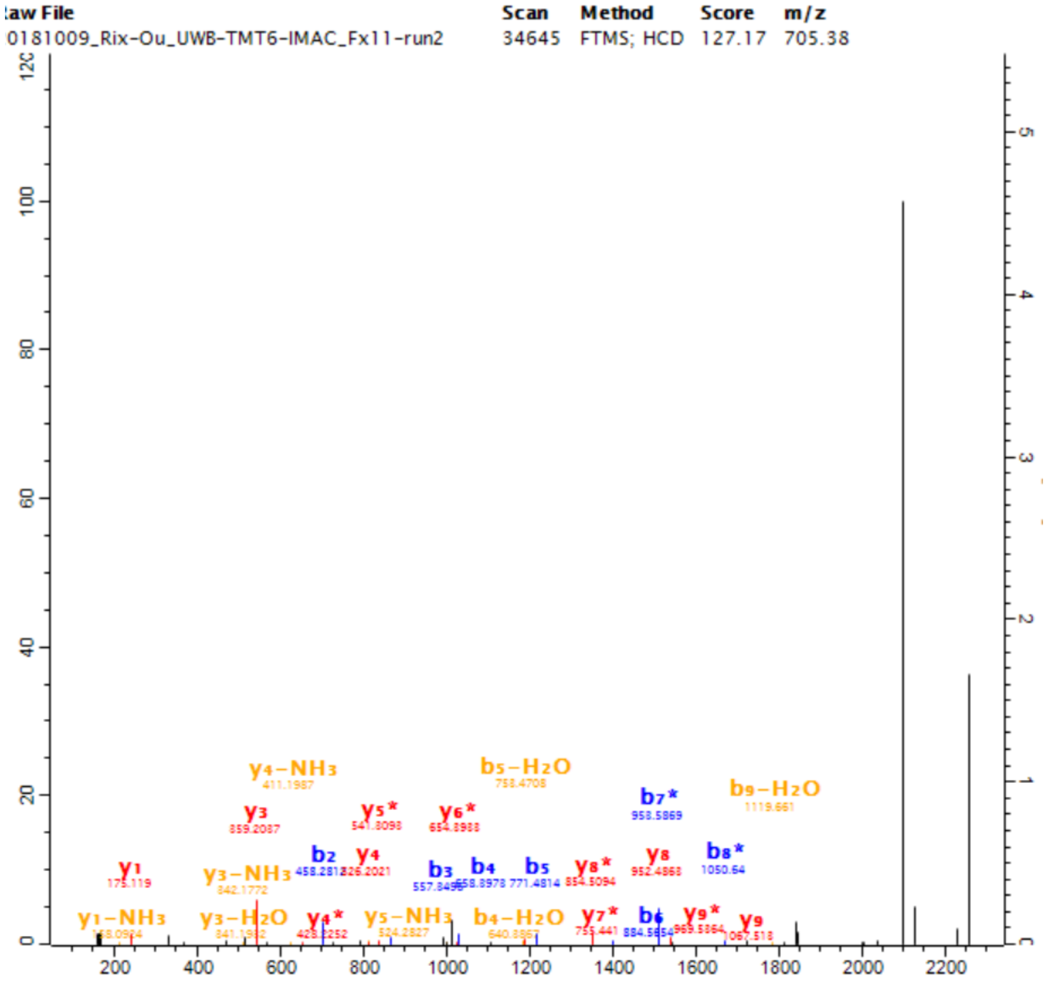

EGFR_Y1197

GSTAENAEY(1)LR

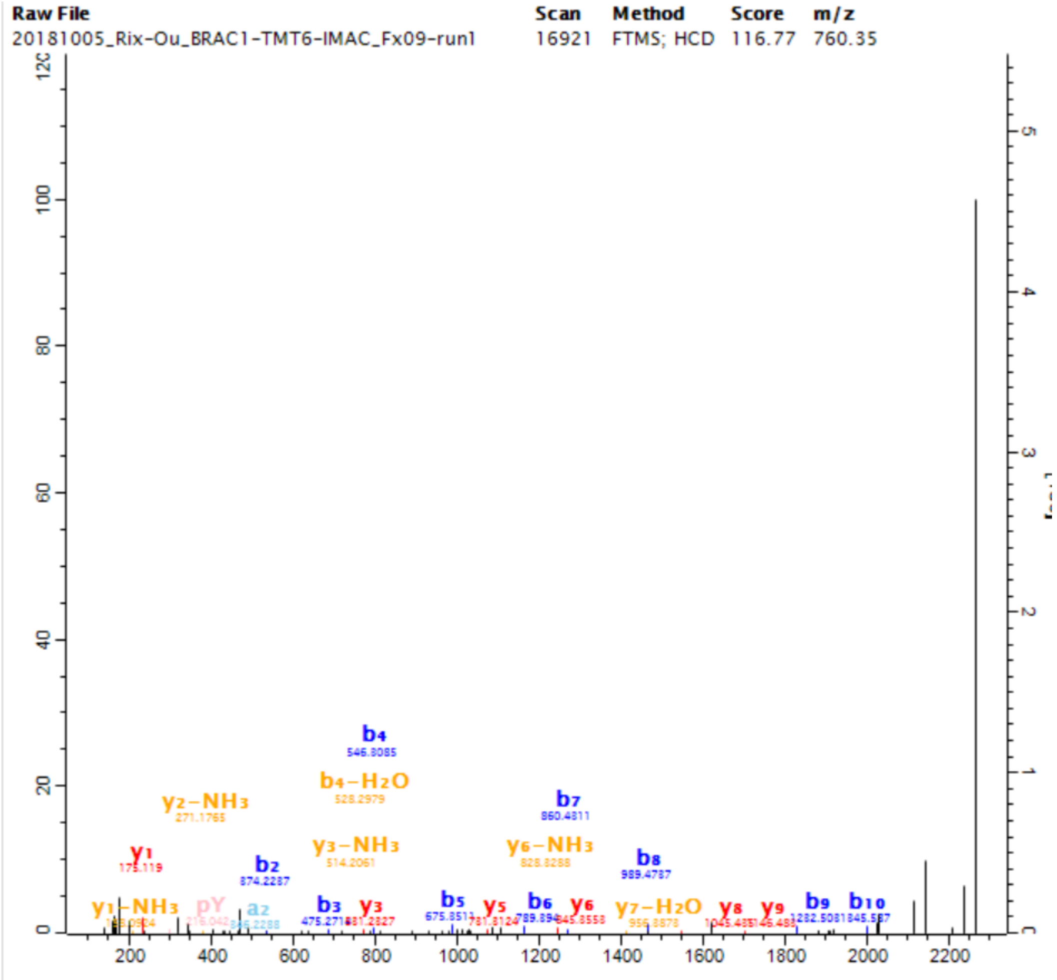

MCM3_S728

T(0.002)ADS(0.997)QET(0.002)KESQKVELSESR

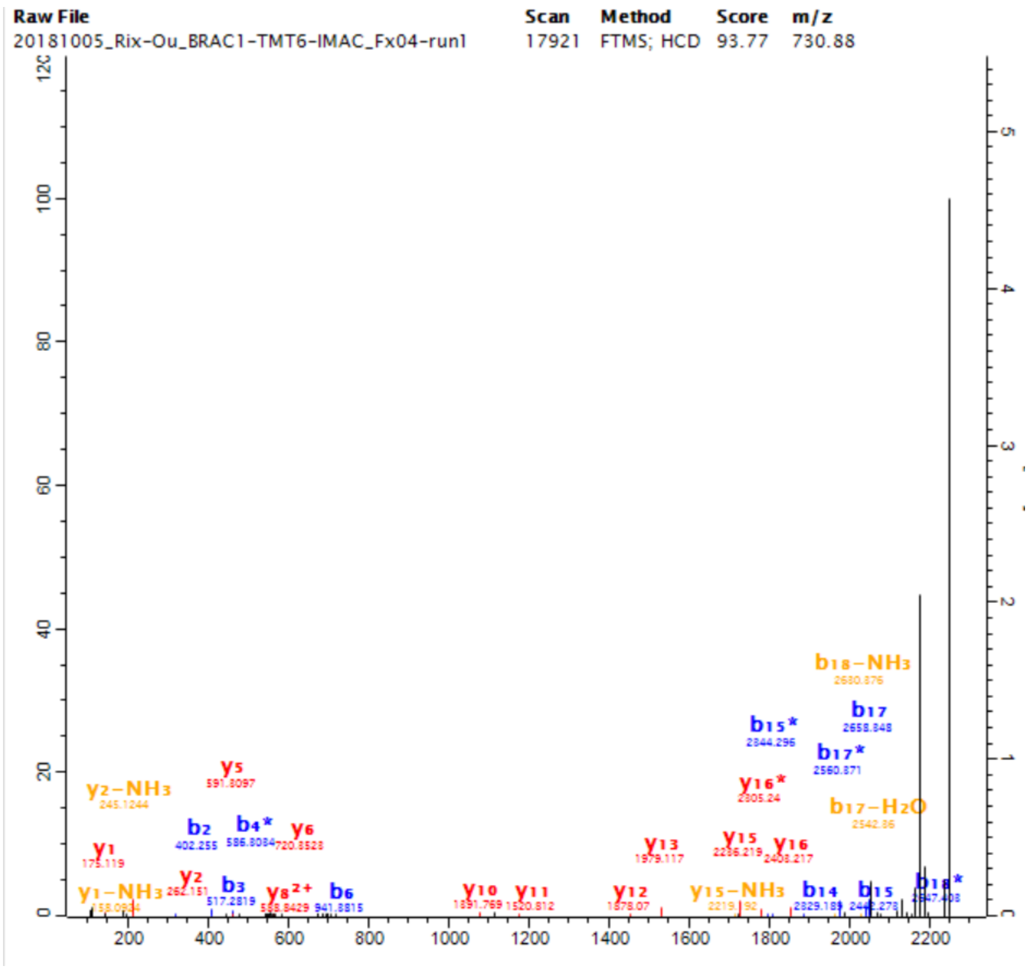

PLK1_T214

T(0.459)LCGT(0.54)PNY(0.001)IAPEVLSK

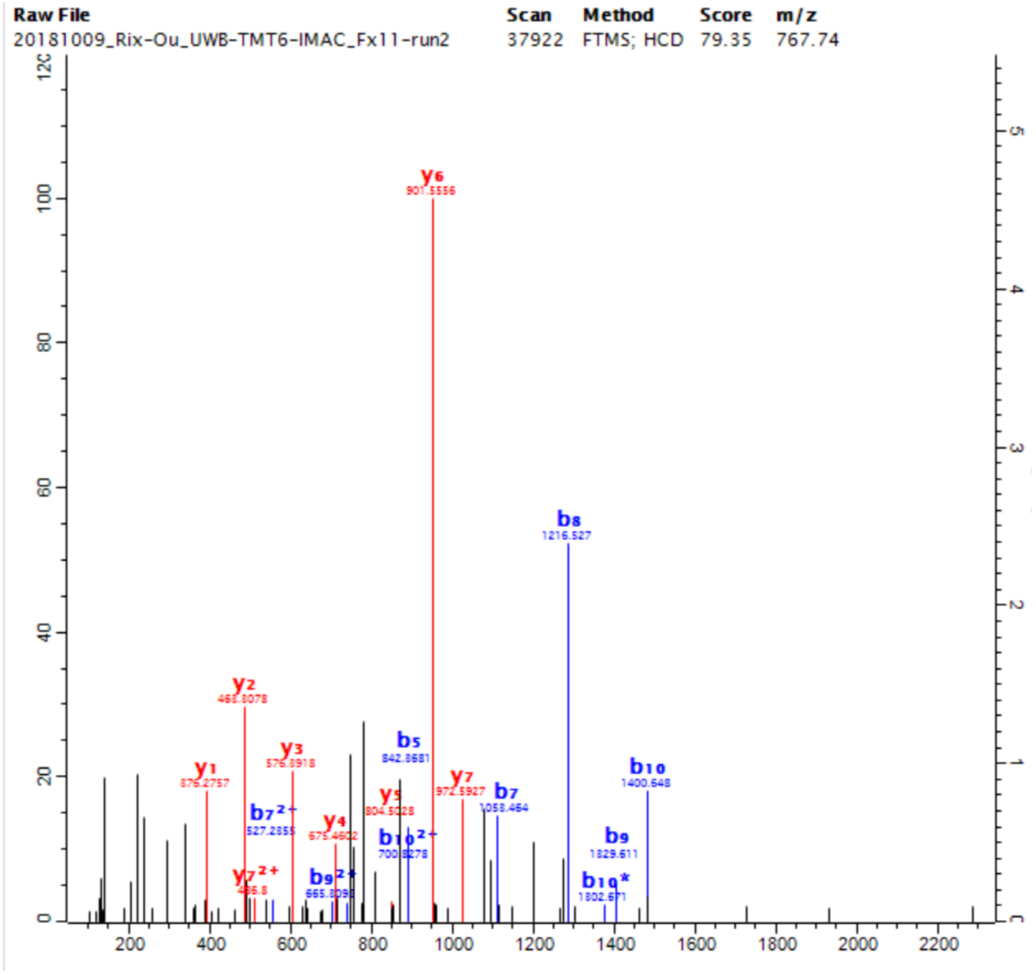

PAK1_S144

Y(0.004)MS(0.996)FT(0.001)DK

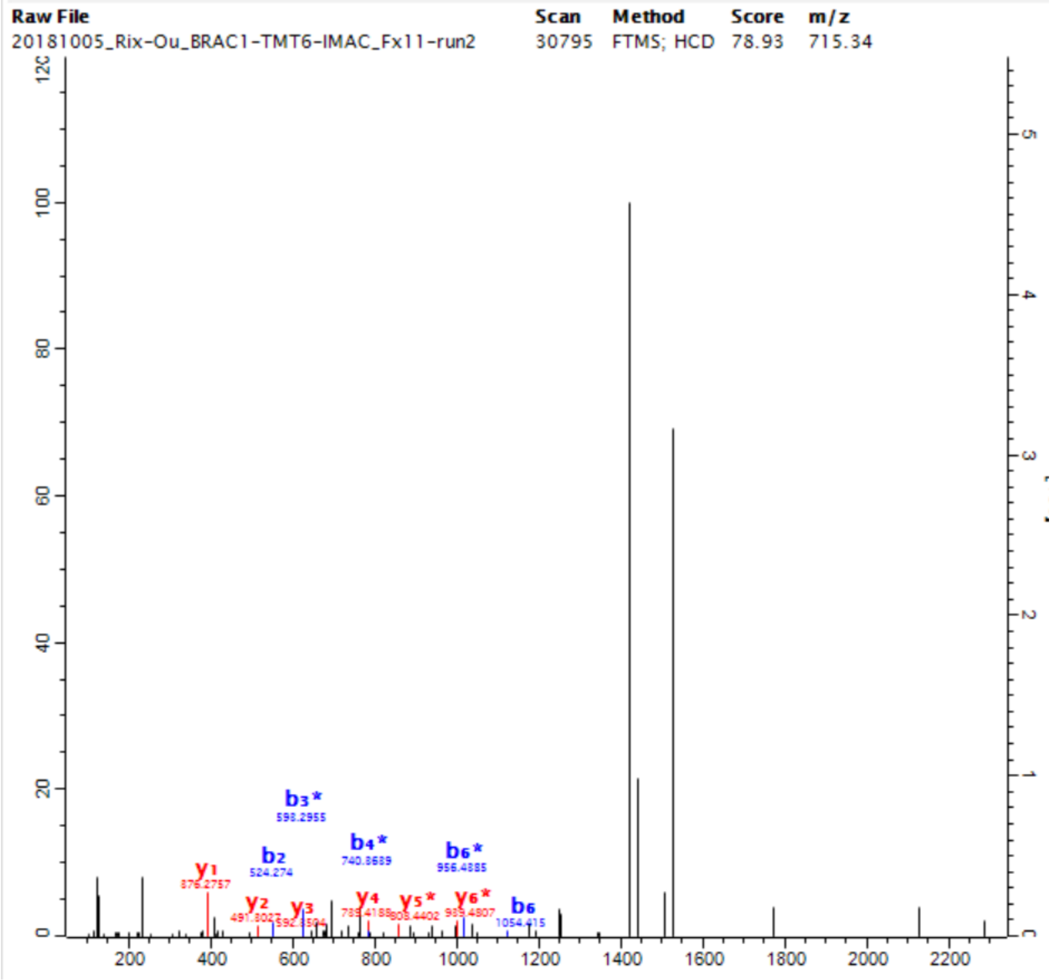

MAPK3_T207;_Y204; _T202

IADPEHDHT(0.007)GFLT(0.593)EY(0.731)VAT(0.669)R

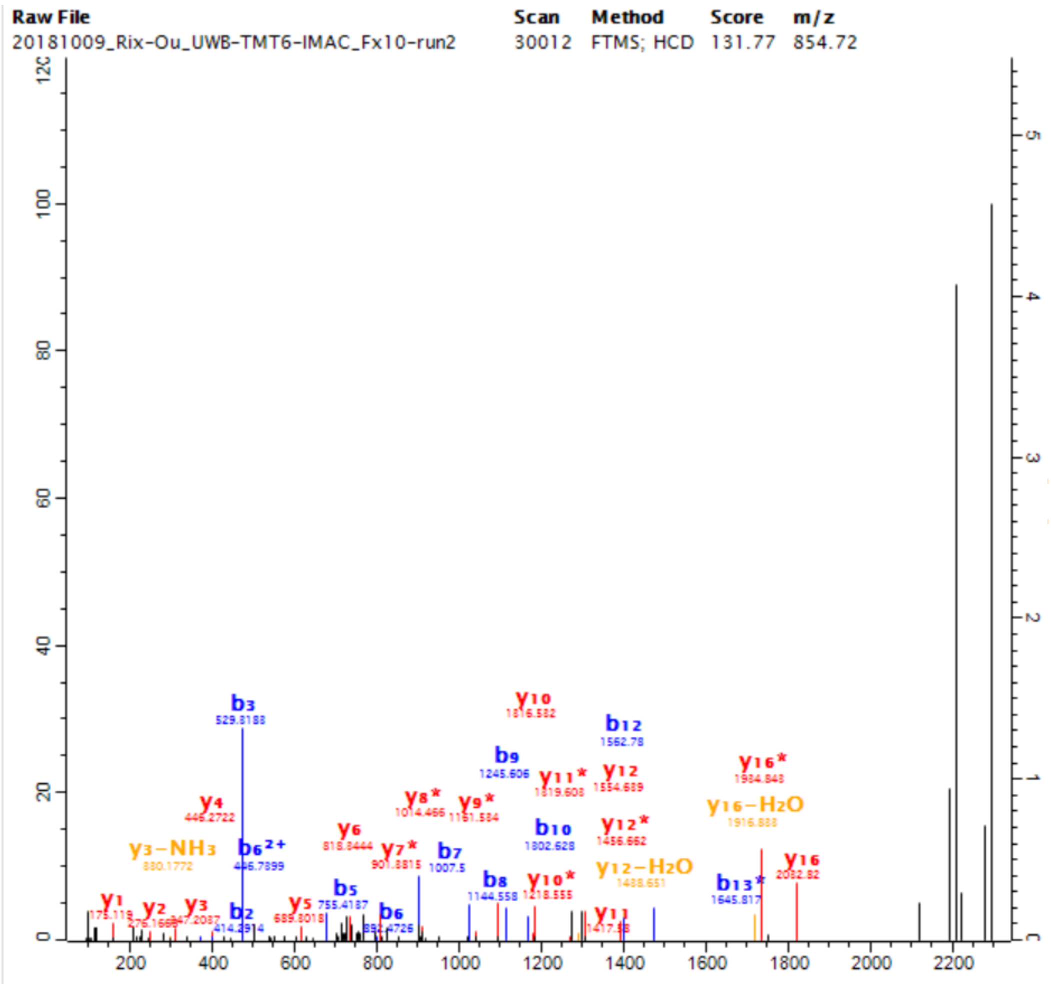

ZYX_S344

S(1)PGAPGPLTLK

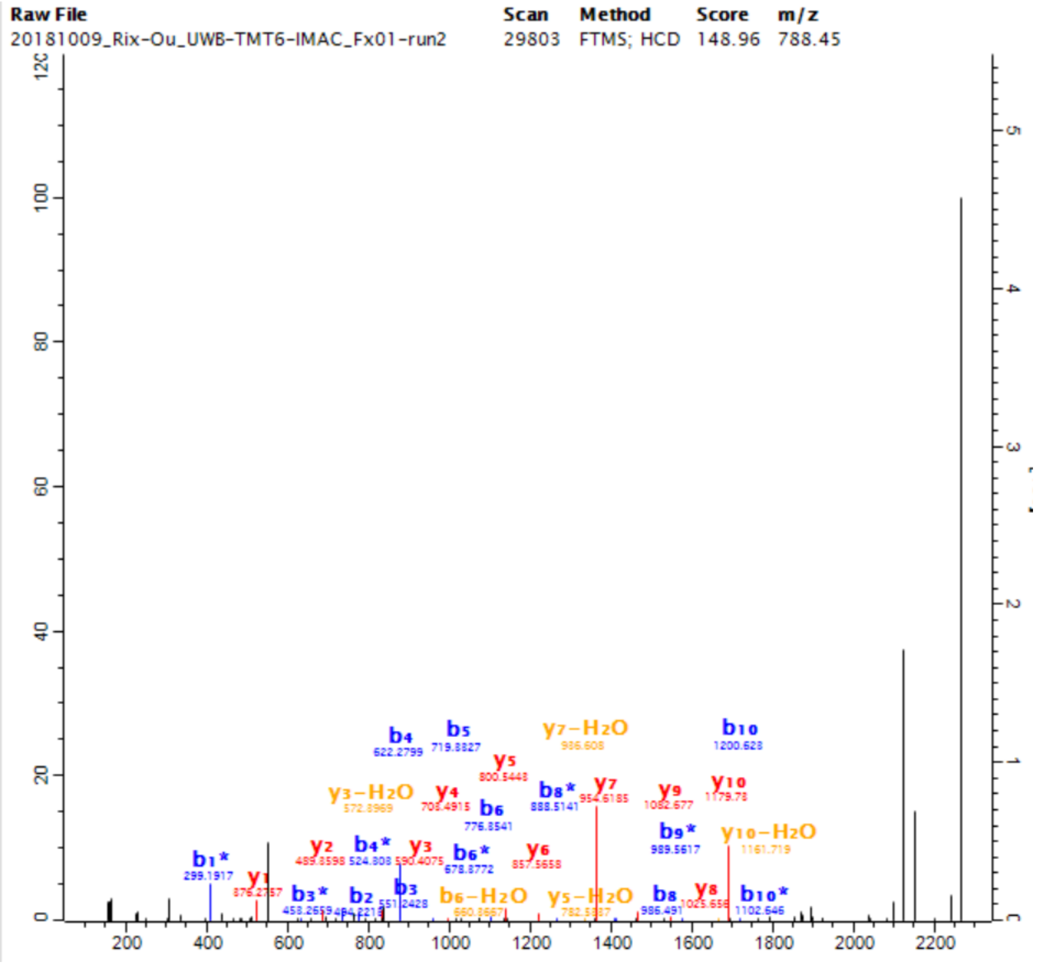

Supplement: Supplemental Figure S2 — MS/MS spectra of the selected differential phospho-peptides depicted inFigure 3D. [file mmc3.pdf]
